# An adaptive, interacting, cluster-based model for predicting the transmission dynamics of COVID-19

**DOI:** 10.1016/j.heliyon.2020.e05722

**Published:** 2020-12-14

**Authors:** R. Ravinder, Sourabh Singh, Suresh Bishnoi, Amreen Jan, Amit Sharma, Hariprasad Kodamana, N.M. Anoop Krishnan

**Affiliations:** a Department of Civil Engineering, Indian Institute of Technology Delhi, Hauz Khas, New Delhi, 110016, India; b Molecular Medicine Group, International Centre for Genetic Engineering and Biotechnology, Aruna Asaf Ali Road, New Delhi, 110 067, India; c Department of Chemical Engineering, Indian Institute of Technology Delhi, Hauz Khas, New Delhi, 110016, India; d Department of Materials Science and Engineering, Indian Institute of Technology Delhi, Hauz Khas, New Delhi, 110016, India

**Keywords:** Microbiology, Computational mathematics, Mathematical modeling, Epidemiology, Public health, Infectious disease, COVID-19, Transmission dynamics, Effective reproduction number, RT

## Abstract

The SARS-CoV-2 driven disease COVID-19 is pandemic with increasing human and monetary costs. COVID-19 has put an unexpected and inordinate degree of pressure on healthcare systems of strong and fragile countries alike. To launch both containment and mitigation measures, each country requires estimates of COVID-19 incidence as such preparedness allows agencies to plan efficient resource allocation and to design control strategies. Here, we have developed a new adaptive, interacting, and cluster-based mathematical model to predict the granular trajectory of COVID-19. We have analyzed incidence data from three currently afflicted countries of Italy, the United States of America, and India. We show that our approach predicts state-wise COVID-19 spread for each country with reasonable accuracy. We show that R_t,_ as the effective reproduction number, exhibits significant spatial variations in these countries. However, by accounting for the spatial variation of R_t_ in an adaptive fashion, the predictive model provides estimates of the possible asymptomatic and undetected COVID-19 cases, both of which are key contributors in COVID-19 transmission. We have applied our methodology to make detailed predictions for COVID19 incidences at the district and state level in India. Finally, to make the models available to the public at large, we have developed a web-based dashboard, namely “Predictions and Assessment of Corona Infections and Transmission in India” (PRACRITI, see http://pracriti.iitd.ac.in), which provides the detailed R_t_ values and a three-week forecast of COVID cases.

## 1 Introduction

Since the first reports from China [1, 2, 3], COVID-19 has spread to all the continents resulting in the infection of more than 1.5 million people and a death toll of more than 100,000 [4,5]. Due to the severity of the pandemic, many countries have implemented complete or partial lockdowns and international travel restrictions [6, 7, 8] to stem disease transmission [9, 10]. As the COVID-19 pandemic presents a very dire economic and humanitarian scenario for most countries worldwide, it is imperative that afflicted governments have ready access to reliable estimates of COVID-19 spread across their states and regions. Such predictive incidence data will enable the deployment of resource allocation strategies, development of new socio-economic policies, and upgradation of healthcare facilities so as to minimize detrimental effects in each country [7, 8, 11].

Several studies have modeled the COVID-19 pandemic at the city, state, or country level [6, 8, 12, 13, 14] using the common Susceptible–Exposed–Infected–Removed (SEIR) model [15], or modifications thereof [16, 17, 18], that can capture the dynamics of an infectious disease such as COVID-19. In the SEIR model, the population is divided into four categories, of which “susceptible” individuals may become “exposed” to the virus through “infected” people who will eventually be “removed” (that is, they can no longer infect others). The removed population refers to the individuals who have recovered or died. The traditional SEIR model when applied to model COVID-19, however, suffers from the following two major limitations: (i) it assumes homogeneity in a large population via keeping the effective reproduction number R_t_ a constant (i.e., local variations in the transmission dynamics within a large population are not accounted for) [15, 19, 20], and (ii) it assumes a “closed population” without demographic variation stemming from births, deaths or migration [15].

China reported its first case on 31 December 2019, with a peak in cumulative cases in an eight-week interval and thence a plateauing. Italy followed the same trajectory after ~11 weeks and then the USA after ~13 weeks (of the first case in China). In India, cases rose after ~12 weeks of the first case in China, and although both cases and deaths are still on the rise in the USA and India, Italy is already witnessing a decrease in daily new cases. To understand the trends of this epidemic, many studies in different countries have employed the R_0_ or R_t_ that was estimated from China. As in other directly contagious diseases, COVID-19 spreads primarily due to human transmission of the pathogen (coronavirus) from city-to-city, or state-to-state, or country-to-country, and this involves significant migration of humans [6, 12, 13]. The dynamics of disease spread, therefore, involves a few primary cases and an index case up to which point the R_t_ is limited in its value. Beyond this, when the infection starts to move from index cases to their contacts, the R_t_ assumes greater magnitude, and then it can drive community transmission that is currently being witnessed in many countries and feared in others that are behind in their epidemic evolution.

**Figure 1:**
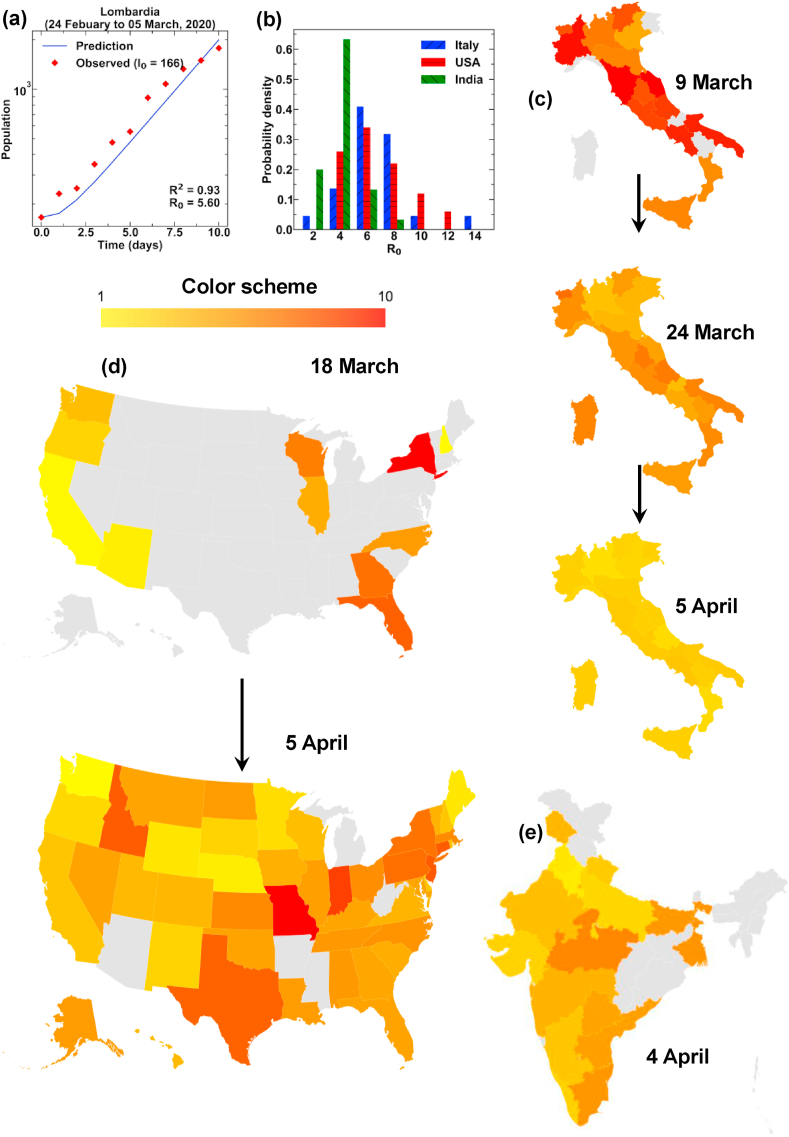
Effective reproduction number R_t_. (a) SEIR model fitted against the observed data (from 24 February 2020 to 9 March 2020) for Lombardia (Italy) to compute its R_t_. Similar approach was applied to all the states for different time periods (see Supplementary Material). (b) Histogram of R_t_ values for Italy (24 February to 9 March), USA (4 March to 18 March), and India (10 March to 24 March) in the early stages of the COVID-19 pandemic. (c) R_t_ in different regions of Italy on 9 March, 24 March and 5 April 2020. (d) R_t_ in different states of the USA on 18 March and 5 April 2020. (e) R_t_ in different states of India on 4 April 2020. The coloring scheme for (c), (d), and (e) is common and is shown in the legend. Grey regions represent the states for which R_t_ cannot be estimated reliably due to the low number of cases.

Although R_t_ is a measure of communicability of COVID-19, its upper range determines the speed of spread. Estimation of R_t_ assumes that everyone around a primary case is equally susceptible to the infection and thereby suggests that it is dependent on the causative agent alone. However, R_t_ is a function of direct and indirect interactions between the agent, host, and environment. The hosts’ immune status, genetic makeup, comorbidities, gender, and smoking can contribute to disease transmission. Equally, the environment that supports transmission is dynamic via variations in temperature, humidity, population density, migration, adaptive interventions like quarantine/isolation/social distancing, socio-economic conditions, and so on [21, 22, 23, 24, 25]. Hence, the use of a constant value for R_t_ at a given time for an entire population, such as a country, cannot capture the evolving transmission dynamics accurately. To address this challenge, we first estimated the spatial variations of R_t_ in Italy, the USA, and India (see Figure 1). Specifically, we tracked COVID-19 spread in each state/region within these countries and then computed R_t_ by explicitly solving the SEIR equations. Interestingly, we did observe that R_t_ exhibited significant spatial variations (see Figure 1), and hence it was deemed inappropriate to be used as a constant, at a given point of time, for any large population. At this point, it should be noted that the R_t_ is calculated from the available data on infected and removed cases. Since this data is fitted with the SEIR equations, the estimate of R_t_ will include the bias in the data and the model. Specifically, the estimation of R_t_ will be affected by the bias in data arising from the sensitivity and specificity of the test, availability of the test kits, and the sampling of the population.

**Figure 2:**
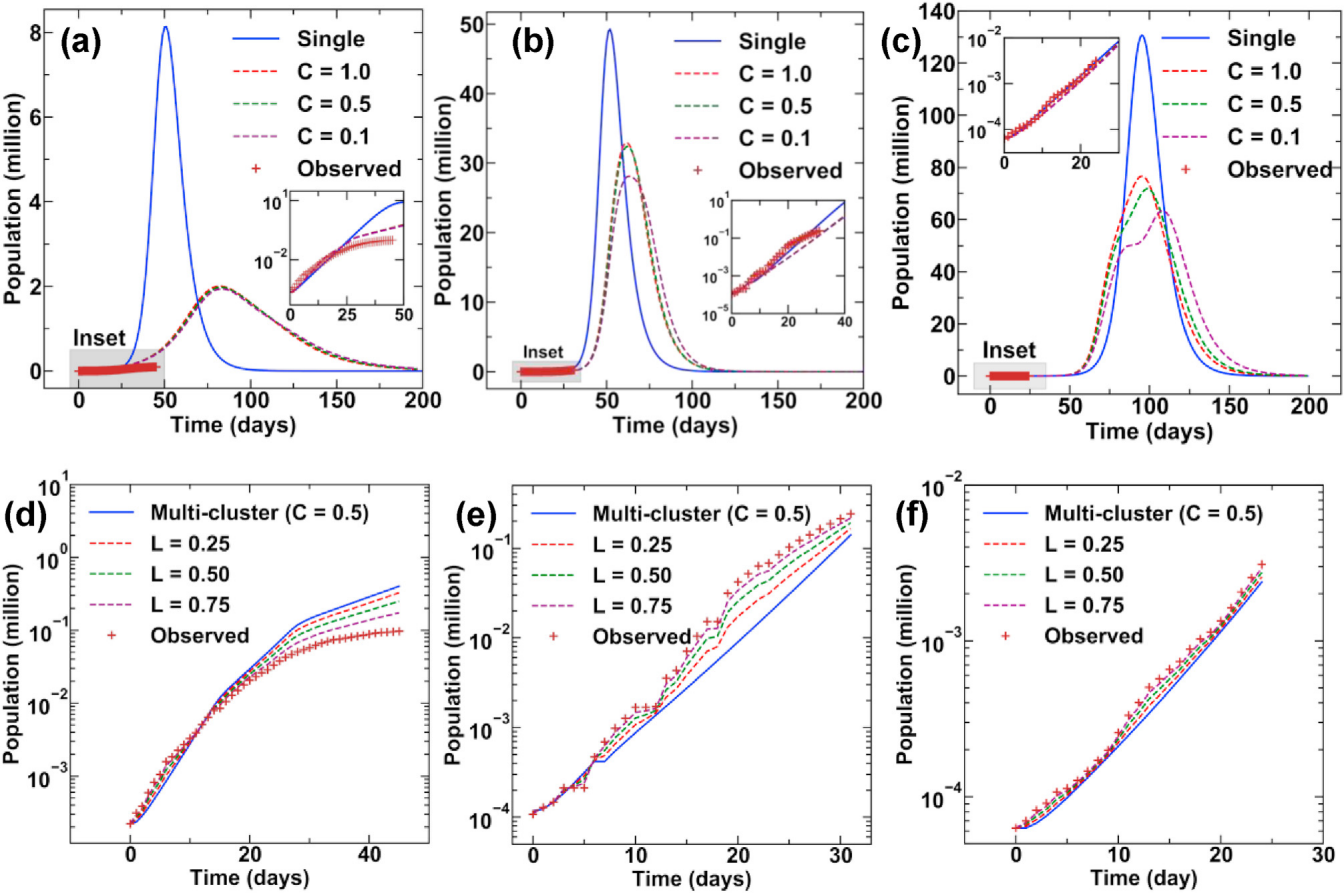
Countrywide spread of COVID-19. Evolution of the pandemic in (a) Italy (b) the USA and (c) India with respect to time. This is based on the traditional SEIR (single cluster) and AICSEIR models with *C* = 1.0, 0.5, 0.1. *C* represents the inter-cluster mobility of the population where *C* = 0 represents zero mobility and *C* = 1 representing restriction-free mobility. INSET for (a), (b), and (c) show fit of model predictions and observed infected cases (square markers). We noted that the variance in comparison to the mean trajectory is significantly small, and it was hence omitted in these figures. The best estimates considering the error between model and observation for (c) Italy, (d) the USA, and (e) India with *L* = 0.25, 0.50, and 0.75. Note that a lower value of *L* suggests increased confidence in the observation, while a higher value of *L* suggests increased confidence in the model. Time *T* = 0 corresponds to 24 February 2020 for Italy, 4 March 2020 for the USA and 10 March 2020 for India.

To address the granularity in R_t_, we used an adaptive, interacting, cluster-based SEIR (AICSEIR) model that, we show, can capture the transmission dynamics of the COVID-19 pandemic within a heterogeneous population (Figure 2). Hereon, the term state represents a subpopulation (or a cluster) in a country. State, therefore, corresponds to the geo-administrative boundaries within India and the USA, and regions in Italy. Our model divided any given country's entire population into multiple, interacting clusters that mingled stochastically. This enabled us to predict the trajectories of COVID-19 transmission in three heterogeneous populations of Italy, the USA, and India up to the state/region level. Typically, R_t_ is estimated by fitting an exponential curve in the early infection stages following the assumption that $I\left(t\right)\approx I\left(0\right)e^{\left(\left[R_{0}-1\right]\gamma t\right)}$. However, due to the paucity of new cases in the early phases, the dynamics can be highly stochastic and influenced by large, noisy fluctuations, which together cause R_t_ estimates to be unreliable [15, 19, 26]. By the time stochastic fluctuations become negligible, the epidemic behavior will tend to be nonlinear due to recoveries or deaths in infected populations rendering the exponential approximation invalid [15]. In such cases, the exponential approach will lead to a significant underestimation of R_t_ due to the removed population (as it is not accounted for in the exponential model). To address these caveats, we computed R_t_ by optimizing predictions from the SEIR model for each state within a country as a function of time (see Methods). This approach is able to capture the time dynamics of R_t_ that emanate as a result of both public health interventions as well as increased infections in a given country.

## 2 Methodology

### 2.1 Dataset

The datasets used for the study include the following. (i) The total number of COVID-19 active and removed cases in three countries—Italy, the USA, and India, along with the state-/region-wise details. These data are obtained from the WHO and the respective government databases [4, 27, 28, 29, 30, 31, 32]. (ii) Population data of each of the states-/regions in the three countries. (iii) Distance between the capital cities of the states in each of the countries is directly calculated from the latitude and longitude of the respective cities. Complete data used in the study are provided in the Supplementary Material (See Supplementary files 2, 3, and 4).

### 2.2 Adaptive interacting cluster-based SEIR (AICSEIR) model

Herein, we present the proposed AICSEIR model (Eq. (1) – Eq. (8)), developed by suitably extending the heterogeneous SIR model [15] that captures the coupling dynamics between populations residing at different geographical locations:

Eq. (1) $$\frac{dX_{ii}}{dt}=\nu_{ii}-\beta_{i}X_{ii}\frac{\sum_{j}Y_{ij}}{\sum_{j}N_{ij}}-C\left(\underset{j}{\sum }l_{ji}X_{ii}+\underset{j}{\sum }r_{ji}X_{ji}\right)-\mu_{ii}X_{ii},$$

Eq. (2) $$\frac{dX_{ij}}{dt}=\nu_{ij}-\beta_{i}X_{ij}\frac{\sum_{j}Y_{ij}}{\sum_{j}N_{ij}}+C\left(l_{ij}X_{jj}-r_{ij}X_{ij}\right)-\mu_{ij}X_{ij},$$

Eq. (3) $$\frac{dW_{ii}}{dt}=\beta_{i}\left(t\right)X_{ii}\frac{\sum_{j}Y_{ij}}{\sum_{j}N_{ij}}-\sigma W_{ii}-C\left(\underset{j}{\sum }l_{ji}W_{ii}+\underset{j}{\sum }r_{ji}W_{ji}\right)-\mu_{ii}W_{ii},$$

Eq. (4) $$\frac{dW_{ij}}{dt}=\beta_{i}\left(t\right)X_{ij}\frac{\sum_{j}Y_{ij}}{\sum_{j}N_{ij}}-\sigma W_{ij}+C\left(l_{ij}W_{jj}-r_{ij}W_{ij}\right)-\mu_{ij}W_{ij},$$

Eq. (5) $$\frac{dY_{ii}}{dt}=\sigma W_{ii}-\gamma Y_{ii}-C\left(\underset{j}{\sum }l_{ji}Y_{ii}+\underset{j}{\sum }r_{ji}Y_{ji}\right)-\mu_{ii}Y_{ii},$$

Eq. (6) $$\frac{dY_{ij}}{dt}=\sigma W_{ij}-\gamma Y_{ij}+C\left(l_{ij}Y_{jj}-r_{ij}Y_{ij}\right)-\mu_{ij}Y_{ij},$$

Eq. (7) $$\frac{dN_{ii}}{dt}=\nu_{ii}-C\left(\underset{j}{\sum }l_{ji}N_{ii}+\underset{j}{\sum }r_{ji}N_{ji}\right)-\mu_{ii}N_{ii},$$

Eq. (8) $$\frac{dN_{ij}}{dt}=\nu_{ij}+C\left(l_{ij}N_{jj}-r_{ij}N_{ij}\right)-\mu_{ij}N_{ij},$$

In the above equations, ‘*i*’ and ‘*j*’ takes values from 1 to *n,* where *n* is the total number of subpopulations. Thus, the values taken by *n* for Italy, the USA, and India are 20, 45, and 30, respectively. $X_{ii},Y_{ii},\;W_{ii},\;N_{ii},\;\nu_{ii},\;\mu_{ii}$denote the number of susceptible, infected, exposed, total hosts, births, and deaths, respectively, in a subpopulation (cluster) ‘*i*’ that live in subpopulation ‘*i*’ and $X_{ij},\;Y_{ij},\;W_{ij},\;N_{ij},\;\nu_{ij},\mu_{ij}$ denote the number of susceptible, infected, exposed, total hosts, births, and deaths in subpopulation ‘*i*’ that live in subpopulation ‘*j*’, respectively. In this study, it is assumed that the number of births and deaths compared to the number of susceptible, infected, exposed, total hosts are negligibly small for the time-period considered and therefore set to zero. The population of host and migrant individuals is computed based on the population of clusters and inter-cluster distance—thus, for two clusters *i* and *j,* the mobility is directly proportional to the population of the cluster *j* and inversely proportional to the distance between *i* and *j*. Thus, two large clusters will have a higher number of exchanges between them as compared to two small clusters. Further, the number of migrants in a given cluster is proportional to its own population—a cluster with a larger population will have a higher number of migrant population. These features have been taken into while initializing $X_{ii}$ and $X_{ij}$ matrices, which represents the number of susceptible in *i* that is originally from *i* and *j*, respectively.

The parameter γ is called the removal or recovery rate, defined as the reciprocal of the average infectious period. In this study, the average infectious period is considered to be three days. $\beta_{i}\left(t\right)$ the parameter indicates the cluster-wise spread of the disease as a function of time. $\beta_{i}\left(t\right)$is evaluated as $\beta_{i}\left(t\right)=\gamma R_{it}$, where $R_{it}$ is the time-dependent effective reproductive ratio of each subpopulation *i*, a key measure that governs the spread of the epidemic. σ parameter is the inverse of the average latent period or average incubation period. In this study, the average incubation period is assumed to be seven days [8, 33].

The variable $l_{ij}$ measures the rate at which individuals leave their home population ‘*j*’ and to subpopulation ‘*i*’, and $r_{ij}$ measures the rate at which individuals leave the subpopulation *‘i’* and to their home population ‘*j*’. We have assumed that during the onset of an epidemic, any individual in the home population would choose to stay there and a fraction of the individuals that live in population ‘*i*’, may return to their home population *‘j’.* Therefore, we have considered $l_{ij}$ to be zero in the model, while $r_{ij}$ is modeled as a stochastic parameter. To this extent, we have assumed that the fraction of the home going migrant population from each subpopulation ‘*j*’ per day will be capped to a fraction ‘*frac*’ of the subpopulation. Hence, the matrix r is generated as a S×S matrix, where Sdenotes the total number of states in a country, with each element $r_{ij}$ is sampled from $r_{ij}∼U\left[0,frac\right]$, where U is the Uniform distribution, with a restriction of $max\left(r_{ij}\right)=frac$. In the study, without loss of generality, *frac* is set to be 0.10. Also note that we have assumed a homogeneous population within a cluster. The total number of people migrated from “*i*” to “*j*” cluster is given by $r_{ij}N_{ij}$. Therefore, the number of susceptible, exposed, infected, and recovered migrated will be given by $r_{ij}X_{ij},\;r_{ij}W_{ij},\;r_{ij}Y_{ij}$, and $r_{ij}Z_{ij}$, respectively, such that $X_{ij}+W_{ij}+Y_{ij}+Z_{ij}=N_{ij}$. It should be noted that although we have assumed the rate of migration of infected and susceptible to be the same, this may not be the case in reality. We have assumed so due to the paucity of any real data. If this data is available, the difference in the migration rate of infected and susceptible individuals can be accounted for in the model by the respective $r_{ij}$ parameter.

Once $r_{ij}$ is frozen, the next step is to calculate $X_{ii}$ and $X_{ij}$. This involves the allocation of the home going migrant population from a native subpopulation to (s−1) other native subpopulations. To this extent, we have assumed that the home of the migrant population is distributed to (s−1) other subpopulations in a ratio directly proportional to the population of the receiver state and inversely proportional to the distance between them. Further, for simplicity, we assume the state capitals are the point of entry and exit points of the migrant population. If we denote $S_{i}$ be the total population of state i, then $X_{ii}=\left(1-r_{ii}\right)S_{i}$ and $X_{ij}=\left(\frac{{\text{a}}_{\text{ij}}}{{\text{b}}_{\text{ij}}}\right){\text{r}}_{\text{ij}}\left(1-S_{i}\right)$, where ${\text{a}}_{\text{ij}}$ is the fraction of the population of the receiver state normalized with the population of remaining (s−1) states and ${\text{b}}_{\text{ij}}$ is the fraction distance between capital cities from the feeder state's capital normalized with distance to the capital cities of the remaining (s−1) states.

The infected population matrix Y is initialized with $Y_{ii}$ is equal to the actual number of cases reported in the state i at the start of the simulation day and $Y_{ij}$ set to zero for all the states. Also, the exposed population matrix W is initialized identically to that of the infected population matrix Y to start the simulation. Further, we add an inter-cluster restriction parameter C to tune the effect of restrictions imposed, as the result of various interventions enforced by the state/central administrations, on the mobility of the migrant population from feeder state to receiver state with *C* = 0 representing zero mobility, and *C* = 1 representing restriction-free mobility.

### 2.3 Computation of R_t_

In this study, $R_{t}$ is computed by directly fitting the observations to the proposed model by minimizing the prediction of infections. The optimization formulation for computing $R_{t}$is given below:

Eq. (9) $${\text{β}}_{i}\left(t\right)=\arg_{{\text{β}}_{i\left(t\right)}}{\left(Y_{ii}-Y_{ii}^{observed}\right)}^{T}Q\left(Y_{ii}-Y_{ii}^{observed}\right)$$

subjectto:(i)Eq(1)−Eq(8)and Eq.(10)

Eq. (11) $$\left(ii\right)\;\text{β}\left(t\right)\in R_{+}$$

here, $Y_{ii},\;Y_{ii}^{observed},\;Q,\;R_{+}$ are infections predicted by the model, observed infections, a suitable weight, and a set of real numbers, respectively. Once ${\text{β}}_{i}\left(t\right)$ is computed for each subpopulation *i*, $R_{it}\;is\;obtained\;as\;\beta_{i}\left(t\right)=\gamma R_{it}$. However, the key point is that due to various interventions of state-wise and country-wise interventions $R_{it}$ would be varying over time. Hence, to make our study realistic, we adaptively re-estimate $R_{it}$ using every 14 days' data by employing Eq. (9)–Eq. (11).

### 2.4 Model correction using real-time observations

It is imperative to reconcile the model predictions of the AICSEIR model with the clinically diagnosed infected case due to the following reasons: (i) Model predictions will be overestimating the total number of infected cases as predictions only depend on $R_{t}$ and the initial infected population. (ii) Clinically diagnosed cases will be underestimating the total number of infected cases due to the testing limits or saturation. Hence, a realistic estimate of the total number of infected cases will be following a middle ground between the two. To this extent, we propose a weighted prediction correction strategy motivated by Kalman filter estimates:

Eq. (12) $$Y^{estimate}\left(t\right)=Y\left(t\right)+L\left(Y^{observed}\left(t\right)-Y\left(t\right)\right)$$

here, $Y^{observed}\left(t\right)$ is the clinically diagnosed infected cases, $Y^{estimate}\left(t\right)$ is a realistic estimate of infected cases, and L is the weighting factor with |L|∈[0,1] and can be tuned based on the real scenarios. *L* value of 0 implies 100% confidence in the model, while an *L* value of 1 implies 100% confidence in the observation [34]. It should be noted that the error in the model prediction may come from both the bias in the model and the data or a combination thereof. The bias in the model comes from various factors that are unaccounted including the quarantined population, effect of social distancing (or wearing masks), variations in the virulence of the virus, variations in the incubation period and recovery period among the population, to name a few. Similarly, the bias in the model comes from several factors such as the sensitivity and the specificity of the tests, availability of test-kits, and effective sampling of the total population. Thus, the role of *L* in the model is a fair attempt to calibrate the AICSEIR model predictions with potentially biased data so as to provide reasonable estimates of predictions for the future. This will also allow one to estimate the number of undetected or asymptomatic cases as the AICSEIR model provides the possible upper limit of the COVID-19 cases.

## 3 Results

### 3.1 Effective reproduction number of COVID-19

To validate our approach, we used the SEIR model to fit actual COVID-19 incidence data for Lombardia of Italy (Figure 1(a), see Methods), and then computed its R_t_ values [4, 27, 28, 29, 30, 31, 32]. The high R^2^ value associated with the fit suggests that the derived R_t_ values are reliable for the time-period considered (Figure 1(a) and Supplementary Material 1). We then proceeded to do this for all the 30 states within India, 45 within the USA, and 20 regions of Italy (Figures 1(b)–(e)). While in few cases, the R^2^ fits were poor due to low initial infection load, most states in the three countries produced reliable R_t_ values (Figures 1(c)–(e) and Supplementary Material 1). It was noted that states with high incidence returned very high R^2^ values, and thus, we considered all R_t_ values with R^2^ > 0.8. For the few other states, R_t_ was assumed to be the country average. Such analyses resulted in a dynamic R_t_ profile for each of the three countries in the early stages of the COVID-19 outbreak (Figure 1(b)). Interestingly, we observed that for both Italy and the USA, the R_t_ values exhibited significantly broader distribution ranging from ~2–14 and ~4–12, respectively (detailed state-wise plots for estimating R_t_ along with the exact R_t_ scores are provided as Supplementary Material 1). On the contrary, in the case of India, we observed that R_t_ values ranged from ~ 2-6 (Figure 1(b)). This evident variation in the ranges of R_t_ values is in congruence with the observed slower rate of early COVID-19 spread in India when compared to the USA and Italy despite the fact that all three countries reported their first COVID-19 case at the end of January 2020.

We next analyzed the temporal variations in R_t_ as it is significantly altered due to many factors, including travel restrictions, state-wise lockdowns (as in parts of the USA), and countrywide lockdown (as for Italy and India). We, therefore, calculated R_t_ for Italy prior to lockdown (that is before 9 March 2020), two weeks into lockdown, and four weeks into lockdown (Figure 1(c)). For the USA, we estimated R_t_ with a two-week interval period (Figure 1(d)). Moreover, in the case of India, due to the delayed onset of the spread of disease, we computed a single R_t_ (Figure 1(e)). These data provide the R_t_ landscape as a choropleth map for each country (Figure 1(c)–(e). As is evident, the R_t_ for Italy decreased significantly due to its lockdown routines (Figure 1(c)). Indeed, enforcement of stricter mobility restrictions has reduced Italian R_t_ values closer to unity, thereby controlling the growth of the epidemic (Figure 1(c)). For the USA, it is clear that only the states that implemented substantial restrictions have managed to reduce their R_t_ values (Figure 1(d)). For India, the strict screening of incoming international travelers and the early imposition of lockdown resulted in reduced R_t_ values in comparison to Italy and the USA. These analyses, therefore, immediately reveal the benefits of public health interventions, and such modeling approaches may be used widely and routinely for the assessment of intervention outcomes.

### 3.2 Adaptive interacting cluster-based SEIR (AICSEIR) model

Based on revised R_t_ profiles, we then used our AICSEIR model (see Methods for details) to predict COVID-19 spread in Italy, the USA, and India. For this, our model required total state population, values of distance between the capital cities of two-states, initial infected number (it could be zero), and the temporal variations in R_t_ (as estimated in the previous section, see Methods). The total population of any state was divided into native and migrant categories (latter was set to 10%). It was assumed that the distribution of a state's migrants was directly proportional to the population of the home state and was inversely proportional to the inter-capital distance. Therefore, two implicit assumptions in these analyses are: (a) people are prone to migration from a highly populated state, and (b) the likelihood of choosing a nearby state for migration is higher. Further, indirect measures of migration, such as airline/train/bus data and the number of tourists, were ignored.

We then compared the trajectories of infection prevalence in Italy, the USA, and India using both the traditional SEIR model (represented as a single in Figure. 2(a)–(c)) and our new AICSEIR model (Figure 2(a)–(c)). A new parameter C was introduced wherein values of 1.0, 0.5, and 0.1 represent the inter-cluster interaction restrictions (C of 0 and 1 denote the absence of migration versus free migration, see Methods for details). All presented models were run extensively with multiple random seed values to account for the stochastic parameter $r_{ij}$ that considers migration as a random event (see Methods). Note that the *r*_*ij*_ values are representative of the migration between different clusters—*r*_*ij*_ measures the rate at which individuals return to their population “*j*” from the population “*i”*. The C value represents the extent of lockdown enforced by a given cluster (C = 0 means no movement between clusters, and C = 1 means no restriction to movements). Thus the value of C will be decided from the enforced lockdown by the state governments, whereas $r_{ij}$ depends on the economic and geographic connectivity between two clusters. The effective migration of a population is given by the product of $r_{ij}$ with C. Using this, a direct comparison of the predictive robustness of SEIR and AICSEIR models in the context of true incidence in the three countries is possible (Figure 2(a)–(c)). We observed SEIR significantly overestimates the peak-infected population (five-fold for Italy and up to 1.8 fold the USA and India). In contrast, the AICSEIR provided a closer estimation of infected cases (Figure 2(a)–(c)). Thus, our approach was able to recapitulate the epidemiological trends reasonably, both on a countrywide scale and its constituent states/regions.

It is noteworthy that the model provides a prediction for total infected, but the observations are based on clinically detected cases. Therefore, both these estimates suffer from the following deficiencies. The clinically detected cases will always underestimate the number of infected cases as the number of tests conducted limits the detection. Besides, all asymptomatic infections shall be missed. On the other hand, our model might still overestimate the total number of cases (but not as much as the SEIR approach) as it is based on the initial conditions and infection dynamics as per R_t_ values. Indeed, there are a host of other confounding factors that can govern R_t_, such as the climatic conditions, host genetics, immune status, age, gender, and comorbidities. Therefore, the best estimate of the total infected population lies between model predictions and actual observation (Figure 2(d)–(f)). While their difference could be small in the early stages, the disparity could be staggering at later stages. To account for this unreliability, we have added a model correction factor *L*, inspired by the Kalman filter that provides an estimate of the infected population [33]. Here, the estimate of the infected population at any time t is computed as the sum of the infected population in the previous timestep t−1 and the difference between observed and model prediction at t weighted with *L* (see Methods). *|L|* resides between 0 and 1 based on the confidence of the model and observation: *L* value of 0 implies 100% confidence in the model, while a value of 1 implies 100% confidence in the observation. We suggest that the former (*L* = 0) can be used in countries with a scarce level of COVID-19 testing, while the latter (*L =* 1) can be used where there is ample testing capacity (Figures 2(d)–(f)). In this scenario, the real observations provide a lower bound of the infected cases, while our AICSEIR model provides the upper bound. This, in turn, allows the estimation of infections that may be undetected or asymptomatic, as both play major roles in the transmission of the infections. It should be noted that if the difference between model prediction and observation is small, that if the error in the model prediction is low, the value of *L* will not have much effect on the pandemic evolution. However, if the difference is large, *L* = 1 will lead to delayed peaking of the total number of COVID incidences, while *L* = 0 will lead to early peaking of the total number of cases. An alternate approach to estimate the value of *L* could be serosurveys, which can be compared independently with the total number of cases in a region to know if the testing is reliable, that is, capable of capturing all the cases (*L = 1*) or not (*L* = 0).

### 3.3 Representative state-wise prediction of COVID-19

**Figure 3:**
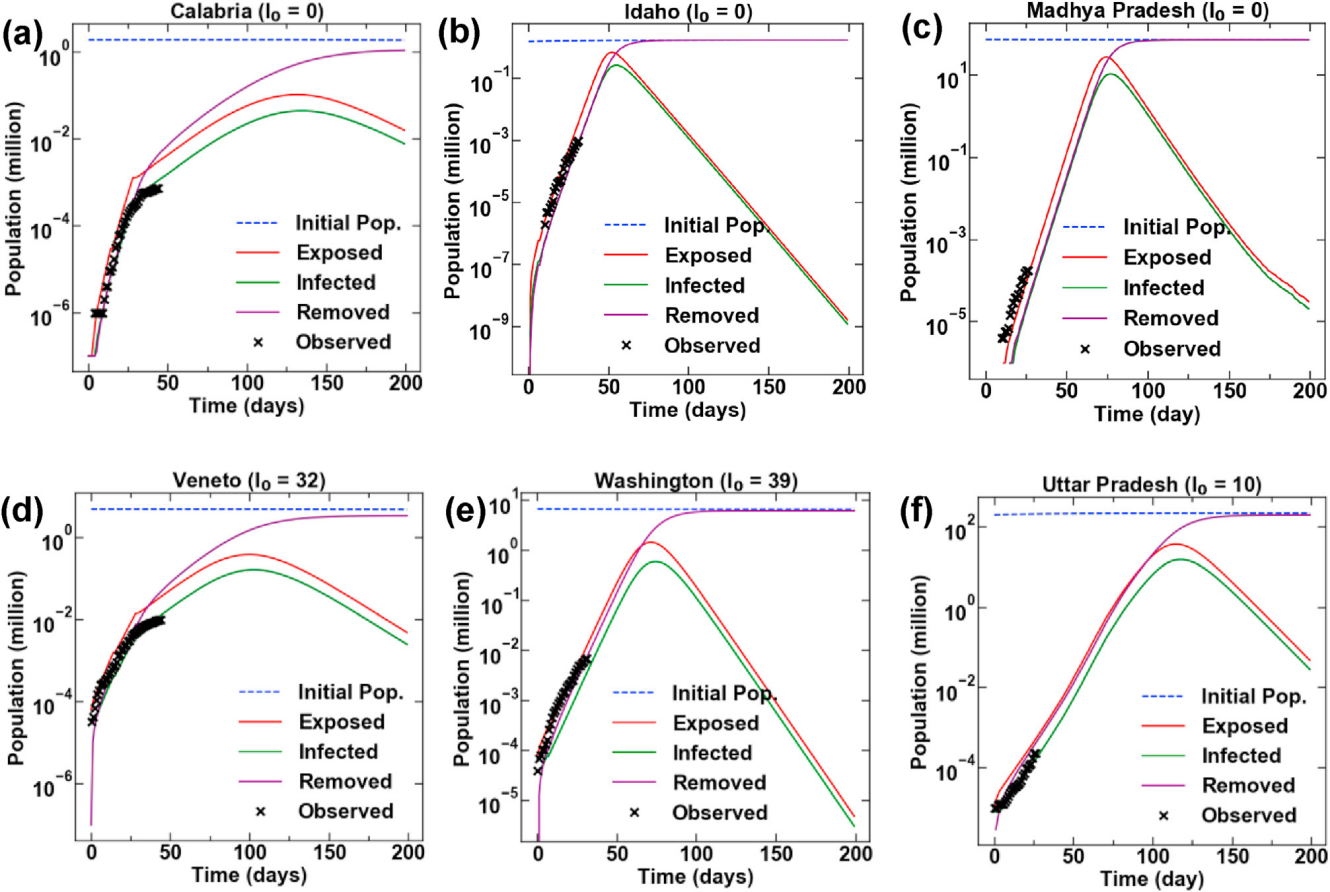
State-wise evolution of COVID-19. Mapping of the pandemic in three states (a) Calabria (Italy), (b) Idaho (the USA), and (c) Madhya Pradesh (India) with zero initial infections as predicted by AICSEIR model in comparison to the observed data. Progression of COVID-19 in three states (d) Veneto (Italy), (e) Washington (the USA), and (f) Uttar Pradesh (India) with non-zero initial infections. It is noteworthy that in both scenarios, our model is able to predict the observed trends to high statistical reliability.

Another facet of our AICSEIR model is its ability to predict the evolution of the infection state-wise or in clusters. Indeed, the country-wise predictions were computed as the summation of sub-populations (state-wise). To validate further, we selected two states from each country and mapped their COVID-19 burden (Figure 3). The initial, exposed, infected, and removed populations of Calabria and Veneto (Italy), Idaho and Washington (USA), Madhya Pradesh, and Uttar Pradesh (India) were assessed (Figure 3). Note that for each country, at least one state chosen had zero initial infected population. For the initiation of infection in these virgin territories, the importation of infected persons would be required based on the cluster interaction term *C* (*C* = 0 would maintain zero infection). We observed that infection trajectories predicted by the model were in agreement with the observed cases for states with zero initial infected population and finite infected population. In other words, through the cluster interaction term, the model is able to realistically predict the spread of COVID-19. We have provided detailed state-wise mapping of populations likely to be infected in the future for each state in each of the countries (30 in India, 45 in the USA, and 20 in Italy, Supplementary Material 3). These data will facilitate state-level and national authorities to devise plans for the allocation of public health resources judiciously at a granularity that addresses state-wise disease burden.

## 4 Discussion

To our knowledge, previous studies on the COVID-19 pandemic have used temporal variations of R_t_ to assess disease spread [6, 8, 12, 35]. We have clearly demonstrated that R_t_ is not constant for a large population and indeed exhibits significant spatial variations. These fluctuations in R_t_ need to be incorporated in the development of realistic epidemiological models. We show the utility of the SEIR model for estimating R_t_, wherein a simple exponential fit may, in the best case, lead to over-/under estimation of R_t_, and in the worst case, may simply be not valid due to the nonlinear variations in disease spread. We show that the temporal variations in R_t_ can be included in an adaptive fashion, while the spatial variations should be included in a granular, cluster-wise model. This approach is capable of capturing the infection dynamics across each nation or, indeed, worldwide. Thus, AICSEIR, with its tunable interaction parameters, can indeed be applied to other infectious diseases.

In addition, the present study provides the following insights into the dynamics of COVID-19 transmission in a multi-cluster population. The spread of the infection amongst multiple clusters is closely associated with the migration of individuals. Thus an uninfected cluster will become infected due to the migration of infected individuals leading to disease spread. Similarly, even if the R_t_ value is low within a cluster, an increased inflow of infected individuals will increase the total cases in a cluster (for e.g., migrant workers coming into a metropolitan city). This might lead to a sudden increase in the cases in a given cluster. Therefore controlling inter-cluster movement is important for reducing disease spread to unaffected and less-affected regions.

At this point, it is worth mentioning some of the limitations of this study: (i) The number of cases reported/detected is closely related to the number of testing per day. The effect of testing needs to be included in the model to account for the asymptomatic and undetected cases. (ii) The migrations between various clusters are presently modeled based on the population and distance between the clusters. Realistic data from transportation networks and travel histories could be used to make migration modeling more quantitative. (iii) In the present study, the migration of susceptible and infected individuals are assumed to be the same. In reality, it is possible that the migration of infected individuals is lower. Such effects could be incorporated into the model based on real data. (iv) The effect of home quarantine and isolation of exposed/infected individuals, respectively, are not included in the present model. (v) Finally, the role of preventive measures such as social distancing, face masks, etc., are not directly included in the model. This is implicitly taken into account through the R_t_.

There are several outcomes of immediate public health value from our work: (i) we provide robust estimates of infection burden with timelines, and this will facilitate proactive development of resource allocation strategies locally [36, 37], (ii) our model provides a caution for regions with low caseload presently as they are likely to follow trends of other highly affected areas in the absence of substantial mobility restrictions, (iii) we suggest a locally graded contextual interventional responses that can factor socio-economic factors and morbidity (note that complete longer-term lockdowns will have notable detrimental economic fallouts resulting in exaggerated impacts on society), (iv) our revised novel coronavirus burden estimates will help map the true extent of infection that includes undetected cases and asymptomatic infections. Although epidemic prediction models tend to discount pivotal contributions from the host and environmental confounders [38, 39], two useful extrapolations of our model are to assess case volumes that may require intensive care and to calculate the true case fatality rates (CFR) [40, 41]. The AICSEIR model can thus serve as a valuable tool for strategizing containment and for stemming mortality associated with the COVID-19 pandemic.

Finally, to make these models accessible to the public, we have developed a web-based interactive dashboard named PRACRITI (PRedictions and Assessment of CoRona Infections and Transmission in India, see: http://pracriti.iitd.ac.in). In the context of India, PRACRITI provides granular data of COVID-19 spread at district-, state- and country-level. Specifically, PRACRITI focuses on two major aspects: (i) predicting the granular R_t_ at district-level and higher and (ii) predicting the highly localized caseload at district-level and higher. It should be noted that the model gives highly accurate predictions for a shorter forecast duration. However, the accuracy decreases as the forecast duration is increased. For this reason, PRACRITI, since its inception in April 2020, provides the forecast for only three weeks forward, which is updated on a weekly basis. To the best of our knowledge, this is the first and only dashboard to provide a detailed, granular distribution of R_t_ values in a country.

## Declarations

### Author contribution statement

R. Ravinder, Sourabh Singh, Suresh Bishnoi, Amreen Jan: Performed the experiments; Analyzed and interpreted the data.

Amit Sharma, Hariprasad Kodamana, N. M. Anoop Krishnan: Conceived and designed the experiments; Analyzed and interpreted the data; Wrote the paper.

### Funding statement

This research did not receive any specific grant from funding agencies in the public, commercial, or not-for-profit sectors.

### Data availability statement

Data included in supplementary material. Data associated with this study has been deposited at GitHub: https://github.com/m3rg-671repo/COVID_modeling.

### Competing interest statement

The authors declare no conflict of interest.

### Additional information

No additional information is available for this paper.

## Appendix A Supplementary data

Sup_1Supplementary file 1: R_t_ values of COVID-19 in all the states/regions of Italy, the USA, and India, along with the figures showing the SEIR fit and the predicted spread of COVID-19 in all the states/regions of Italy, the USA, and India based on the AICSEIR model.

Sup_2 Supplementary file 2: Data on the spread of COVID-19 in Italy, the USA, and India used for estimating Rt and model development. Sup_2

Sup_3 Supplementary file 3: Data on the population of each of the states/regions in Italy, the USA, and India. Sup_3

Sup_4 Supplementary file 4: Data on the coordinates of the capital cities of the states/regions in Italy, the USA, and India. Sup_4
