# Supplementary material for: An adaptive, interacting, cluster-based model for predicting the transmission dynamics of COVID-19
Source: Heliyon. 2020 Dec 14;6(12):e05722. doi: 10.1016/j.heliyon.2020.e05722 (PMC7749387; doi:10.1016/j.heliyon.2020.e05722)
Supplement: Supplementary file 1 — Sup_1Supplementary file 1: Rt values of COVID-19 in all the states/regions of Italy, the USA, and India, along with the figures showing the SEIR fit and the predicted spread of COVID-19 in all the states/regions of Italy, the USA, and India based on the AICSEIR model. [file mmc1.pdf]

An Adaptive, Interacting, Cluster-Based Model For Predicting the Transmission Dynamics of COVID-19

R. Ravinder[1], Sourabh Singh[1], Suresh Bishnoi[1], Amreen Jan[1], Amit Sharma[2,*], Hariprasad Kodamana[3,*], N. M. Anoop Krishnan[1,4,*]

[1]Department of Civil Engineering, Indian Institute of Technology Delhi, Hauz Khas, New Delhi 110016

[2]Molecular Medicine Group, International Centre for Genetic Engineering and Biotechnology, Aruna Asaf Ali Road, New Delhi, 110 067, India

[3]Department of Chemical Engineering, Indian Institute of Technology Delhi, Hauz Khas, New Delhi 110016, India

[4]Department of Materials Science and Engineering, Indian Institute of Technology Delhi, Hauz Khas, New Delhi 110016, India

[*]**Corresponding Authors**: A. Sharma (amit.icgeb@gmail.com), H. Kodamana (kodamana@iitd.ac.in), N. M. A. Krishnan (krishnan@iitd.ac.in).

Supplementary Information

Effective reproduction number of COVID-19

Table S1: Effective reproduction number for states of Italy. SEIR model fitted against the observed data (from 24 February 2020 to 9 March 2020).

State	R_t	R^2
Puglia	8.37	0.99
Toscana	8.74	0.98
Lazio	7.48	0.94
Sicilia	5.22	0.94
Lombardia	5.60	0.93
Abruzzo	7.74	0.92
Piemonte	8.62	0.91
Veneto	3.73	0.91
Emilia-Romagna	5.20	0.90
Campania	8.29	0.89
Calabria	4.97	0.89
Marche	11.49	0.87
P.A. Bolzano	5.23	0.83

Table S2: Effective reproduction number for states of Italy. SEIR model fitted against the observed data (from 9 March 2020 to 23 March 2020).

State	R_t	R^2
Piemonte	4.79	0.99
Valle d'Aosta	6.07	0.98
Abruzzo	5.63	0.98
Emilia-Romagna	3.14	0.97
Umbria	5.60	0.97
Sardegna	5.28	0.97
Campania	3.72	0.97
Puglia	5.28	0.96
Toscana	4.59	0.95
Basilicata	4.34	0.94
Friuli Venezia Giulia	3.11	0.94
P.A. Bolzano	4.92	0.94
Veneto	3.24	0.94
Lazio	4.91	0.93
Calabria	4.98	0.91
Liguria	3.93	0.91
Molise	2.91	0.91
Sicilia	4.30	0.90
Lombardia	2.71	0.86
Marche	4.07	0.86
P.A. Trento	4.32	0.84

Table S3: Effective reproduction number for states of Italy. SEIR model fitted against the observed data (from 23 March 2020 to 9 April 2020).

State	R_t	R^2
Molise	2.37	0.95
Campania	2.49	0.91
Abruzzo	1.55	0.91
P.A. Trento	1.91	0.89
Liguria	1.77	0.86
Puglia	2.33	0.81

Table S4: Effective reproduction number for states of USA. SEIR model fitted against the observed data (from 4 March 2020 to 18 March 2020).

State	R_t	R^2
Arizona	3.96	0.93
California	3.65	0.98
Florida	8.04	0.96
Georgia	7.61	0.96
Illinois	5.97	0.96
New Hampshire	3.57	0.95
New York	10.32	0.96
North Carolina	6.48	0.94
Oregon	4.81	0.85
Washington	5.46	0.94
Wisconsin	7.27	0.98

Table S5: Effective reproduction number for states of USA. SEIR model fitted against the observed data (from 18 March 2020 to 9 April 2020).

State	R_t	R^2
Alabama	5.41	0.87
Alaska	5.34	0.86
California	4.35	0.96
Colorado	4.72	0.94
Connecticut	6.67	0.94
Delaware	4.41	0.96
Florida	5.17	0.97
Georgia	5.10	0.92
Hawaii	4.66	0.87
Idaho	6.65	0.94
Illinois	5.28	0.92
Indiana	7.12	0.91
Iowa	4.88	0.96
Kansas	5.74	0.91
Kentucky	5.13	0.92
Louisiana	5.32	0.89
Maine	3.54	0.93
Maryland	5.14	0.96
Massachusetts	5.69	0.94
Minnesota	3.79	0.90
Montana	5.16	0.91
Nebraska	3.38	0.98
Nevada	5.20	0.95
New Hampshire	4.36	0.95
New Jersey	6.72	0.90
New Mexico	4.04	0.96
North Carolina	5.53	0.90
North Dakota	5.33	0.85
Ohio	5.65	0.90
Oklahoma	5.18	0.92
Oregon	3.92	0.96
Pennsylvania	6.29	0.94
Rhode Island	4.50	0.98
South Carolina	4.98	0.92
South Dakota	4.04	0.97
Tennessee	5.43	0.84
Texas	6.53	0.87
Utah	4.67	0.90
Vermont	4.85	0.85
Virginia	4.78	0.97
Washington	3.03	0.95
Wisconsin	4.54	0.84
Wyoming	3.55	0.96

Table S6: Effective reproduction number for states of India. SEIR model fitted against the observed data (from 10 March 2020 to 4 April 2020).

State	R_t	R^2
Rajasthan	3.50	0.99
Madhya Pradesh	5.64	0.98
Punjab	1.89	0.97
Uttar Pradesh	2.57	0.97
Jammu and Kashmir	3.83	0.96
Haryana	1.71	0.96
Telangana	4.59	0.96
Maharashtra	3.99	0.96
Kerala	2.77	0.96
Tamil Nadu	5.07	0.95
Karnataka	3.22	0.94
West Bengal	5.13	0.93
Chandigarh	2.43	0.93
Andhra Pradesh	4.73	0.91
Delhi	3.98	0.90
Gujarat	2.73	0.90
Bihar	5.02	0.90
Puducherry	7.18	0.88
Uttarakhand	3.00	0.86

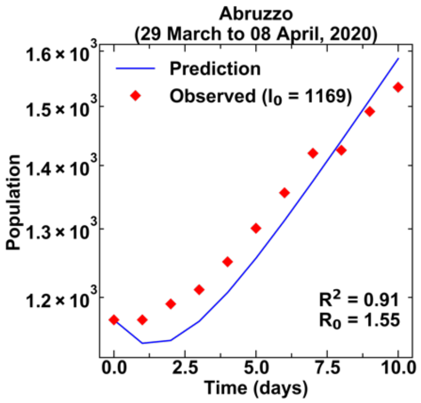

(1)

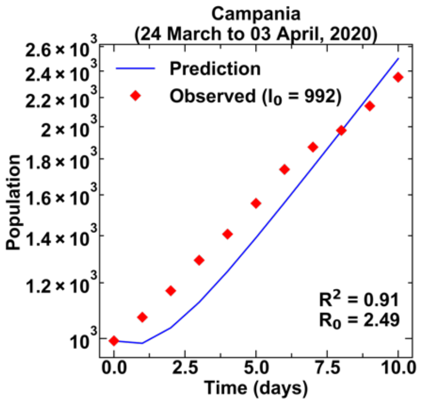

(2)

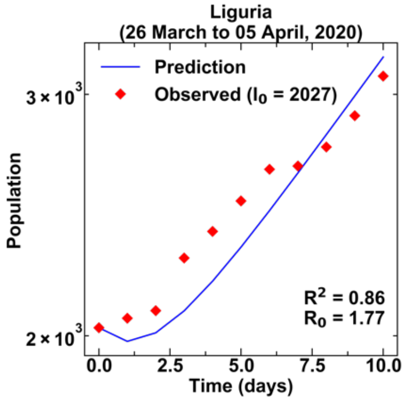

(3)

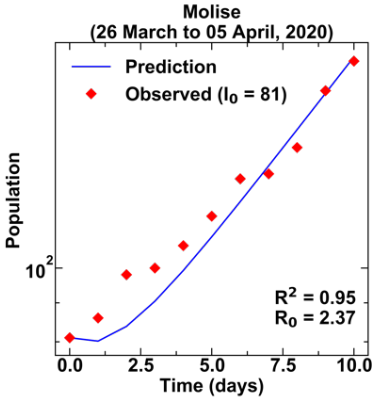

(4)

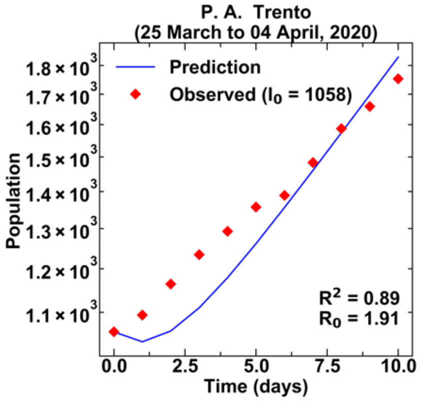

(5)

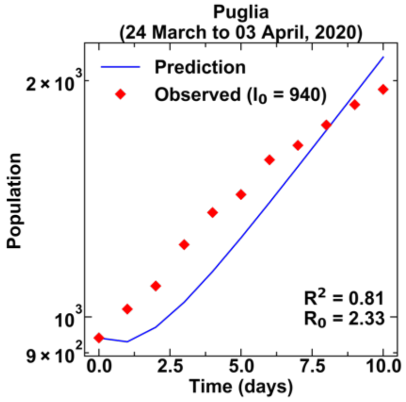

(6)

Fig. S1: SEIR model fitted against the observed data (from 24 February 2020 to 9 March 2020) for all the states of Italy.

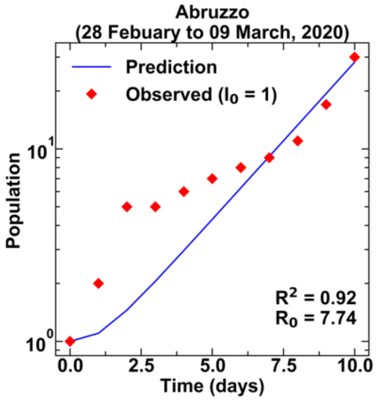
Abruzzo
(28 Febuary to 09 March, 2020)
Prediction
Observed (I_0 = 1)
R^2 = 0.92
R_0 = 7.74
Population
Time (days)

(1)

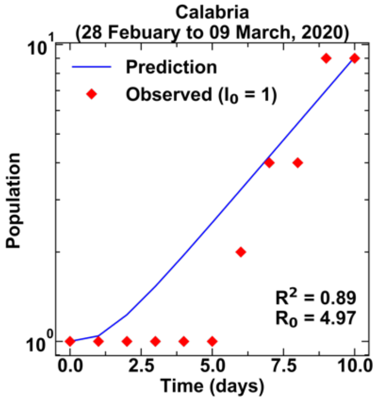
Calabria
(28 Febuary to 09 March, 2020)
Prediction
Observed (I_0 = 1)
R^2 = 0.89
R_0 = 4.97
Population
Time (days)

(2)

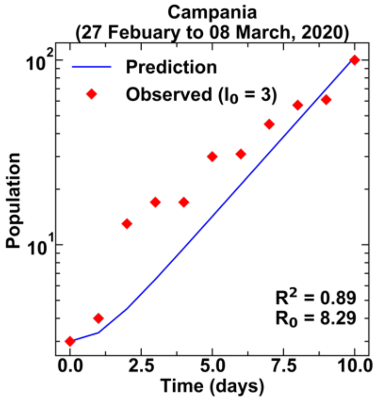
Campania
(27 Febuary to 08 March, 2020)
Prediction
Observed (I_0 = 3)
R^2 = 0.89
R_0 = 8.29
Population
Time (days)

(3)

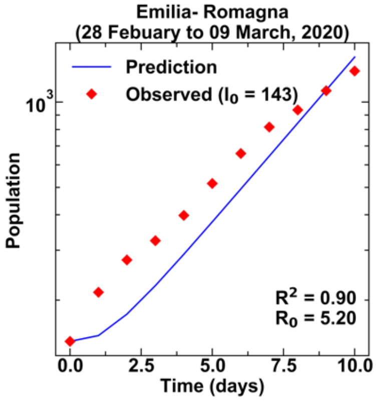
Emilia- Romagna
(28 Febuary to 09 March, 2020)
Prediction
Observed (I_0 = 143)
R^2 = 0.90
R_0 = 5.20
Population
Time (days)

(4)

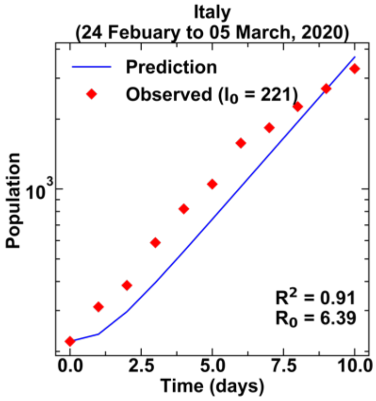
Italy
(24 Febuary to 05 March, 2020)
Prediction
Observed (I_0 = 221)
R^2 = 0.91
R_0 = 6.39
Population
Time (days)

(5)

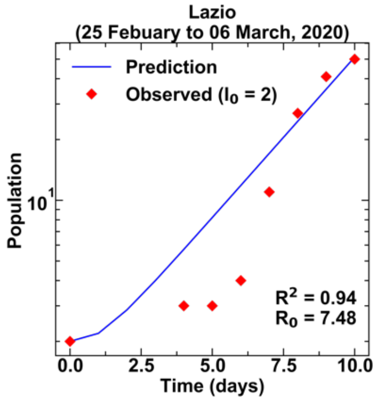
Lazio
(25 Febuary to 06 March, 2020)
Prediction
Observed (I_0 = 2)
R^2 = 0.94
R_0 = 7.48
Population
Time (days)

(6)

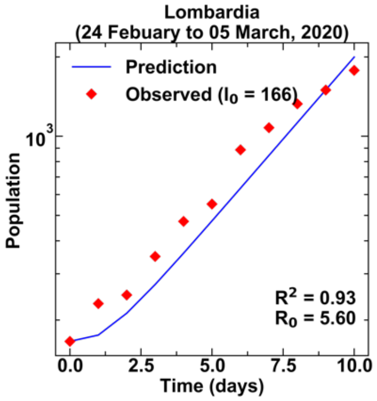
Lombardia
(24 Febuary to 05 March, 2020)
Prediction
Observed (I_0 = 166)
R^2 = 0.93
R_0 = 5.60
Population
Time (days)

(7)

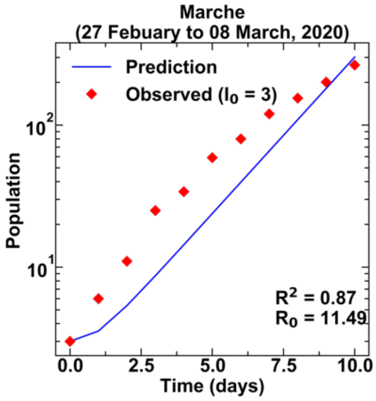
Marche
(27 Febuary to 08 March, 2020)
Prediction
Observed (I_0 = 3)
R^2 = 0.87
R_0 = 11.49
Population
Time (days)

(8)

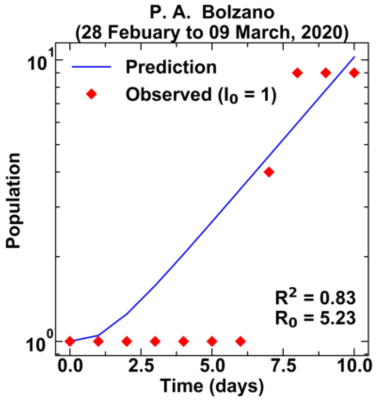
P. A. Bolzano
(28 Febuary to 09 March, 2020)
Prediction
Observed (I_0 = 1)
R^2 = 0.83
R_0 = 5.23
Population
Time (days)

(9)

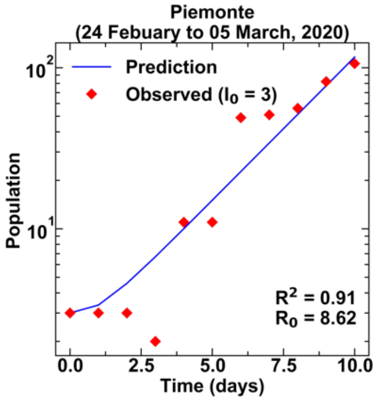
Piemonte
(24 Febuary to 05 March, 2020)
Prediction
Observed (I_0 = 3)
R^2 = 0.91
R_0 = 8.62
Population
Time (days)

(10)

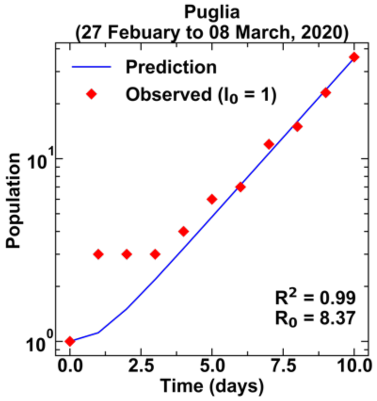
Puglia
(27 Febuary to 08 March, 2020)
Prediction
Observed (I_0 = 1)
R^2 = 0.99
R_0 = 8.37
Population
Time (days)

(11)

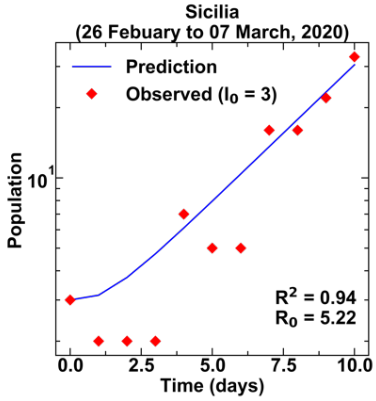
Sicilia
(26 Febuary to 07 March, 2020)
Prediction
Observed (I_0 = 3)
R^2 = 0.94
R_0 = 5.22
Population
Time (days)

(12)

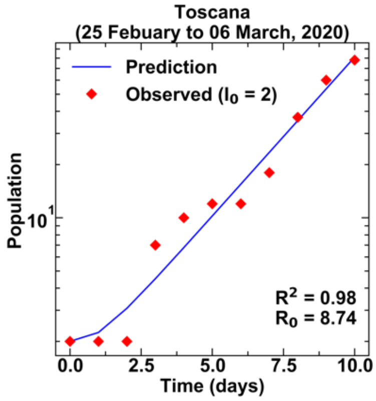

(13)

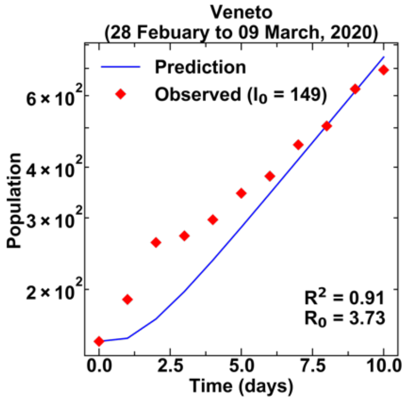

(14)

Fig. S2: SEIR model fitted against the observed data (from 9 March 2020 to 23 March 2020) for all the states of Italy.

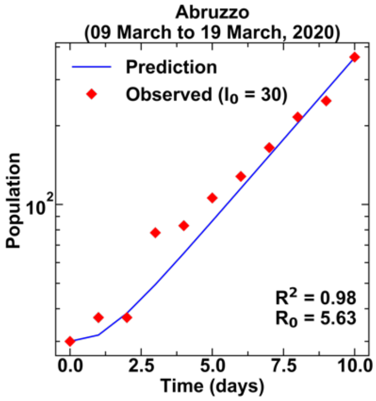
Abruzzo
(09 March to 19 March, 2020)
Prediction
Observed (I_0 = 30)
Population
10^2
$R^2 = 0.98$
$R_0 = 5.63$
0.0 2.5 5.0 7.5 10.0
Time (days)

(1)

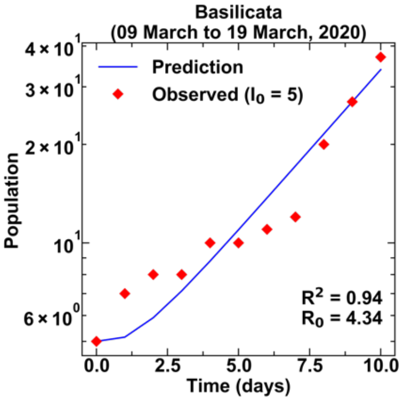
Basilicata
(09 March to 19 March, 2020)
Prediction
Observed (I_0 = 5)
Population
4×10^1
3×10^1
2×10^1
10^1
6×10^0
$R^2 = 0.94$
$R_0 = 4.34$
0.0 2.5 5.0 7.5 10.0
Time (days)

(2)

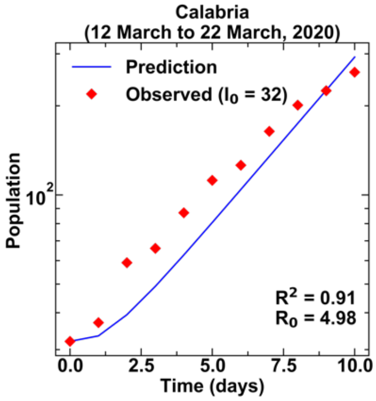
Calabria
(12 March to 22 March, 2020)
Prediction
Observed (I_0 = 32)
Population
10^2
$R^2 = 0.91$
$R_0 = 4.98$
0.0 2.5 5.0 7.5 10.0
Time (days)

(3)

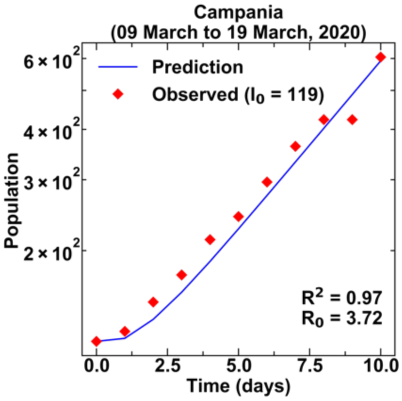
Campania
(09 March to 19 March, 2020)
Prediction
Observed (I_0 = 119)
Population
6×10^2
4×10^2
3×10^2
2×10^2
$R^2 = 0.97$
$R_0 = 3.72$
0.0 2.5 5.0 7.5 10.0
Time (days)

(4)

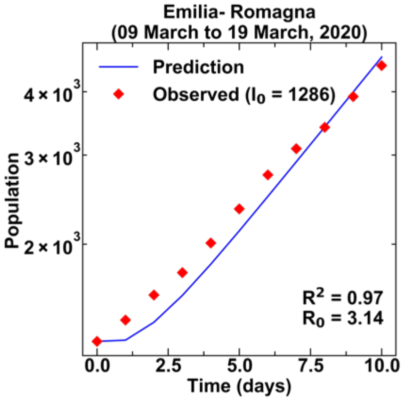
Emilia- Romagna
(09 March to 19 March, 2020)
Prediction
Observed (I_0 = 1286)
Population
4×10^3
3×10^3
2×10^3
$R^2 = 0.97$
$R_0 = 3.14$
0.0 2.5 5.0 7.5 10.0
Time (days)

(5)

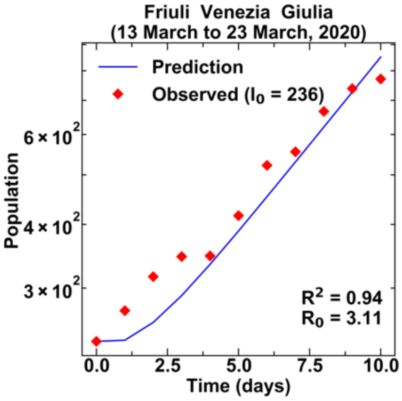
Friuli Venezia Giulia
(13 March to 23 March, 2020)
Prediction
Observed (I_0 = 236)
Population
6×10^2
4×10^2
3×10^2
$R^2 = 0.94$
$R_0 = 3.11$
0.0 2.5 5.0 7.5 10.0
Time (days)

(6)

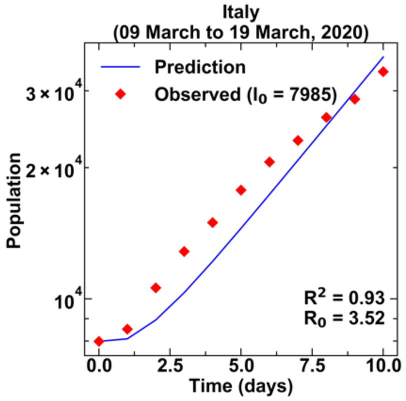
Italy
(09 March to 19 March, 2020)
Prediction
Observed (I_0 = 7985)
R^2 = 0.93
R_0 = 3.52
Population
Time (days)

(7)

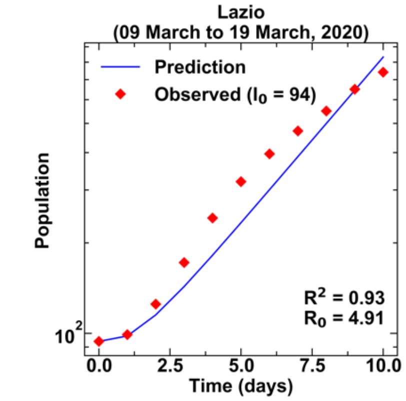
Lazio
(09 March to 19 March, 2020)
Prediction
Observed (I_0 = 94)
R^2 = 0.93
R_0 = 4.91
Population
Time (days)

(8)

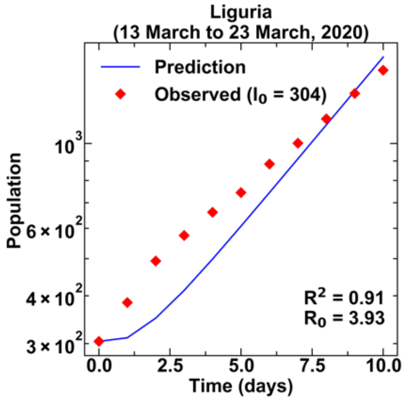
Liguria
(13 March to 23 March, 2020)
Prediction
Observed (I_0 = 304)
R^2 = 0.91
R_0 = 3.93
Population
Time (days)

(9)

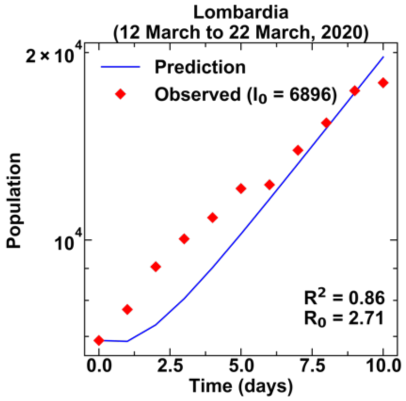
Lombardia
(12 March to 22 March, 2020)
Prediction
Observed (I_0 = 6896)
R^2 = 0.86
R_0 = 2.71
Population
Time (days)

(10)

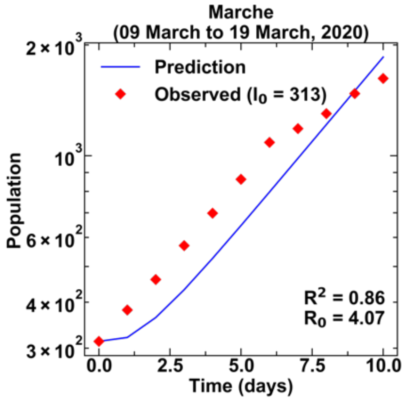
Marche
(09 March to 19 March, 2020)
Prediction
Observed (I_0 = 313)
R^2 = 0.86
R_0 = 4.07
Population
Time (days)

(11)

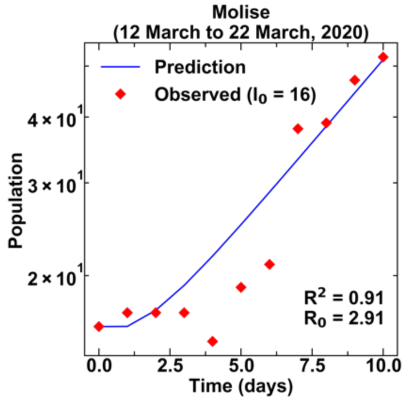
Molise
(12 March to 22 March, 2020)
Prediction
Observed (I_0 = 16)
R^2 = 0.91
R_0 = 2.91
Population
Time (days)

(12)

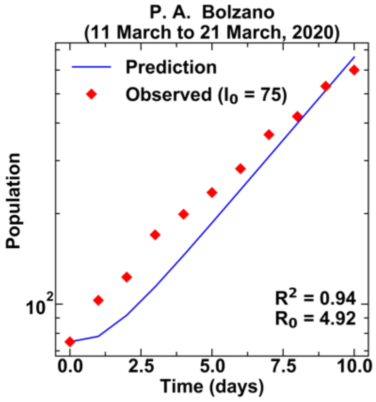

(13)

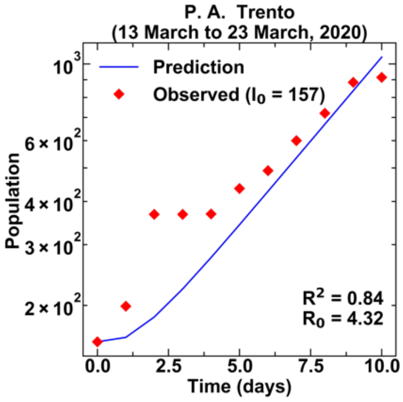

(14)

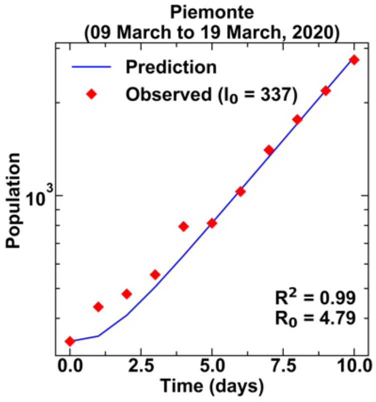

(15)

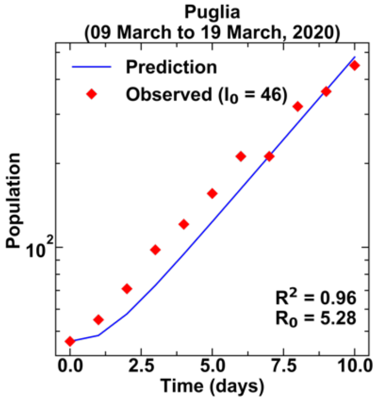

(16)

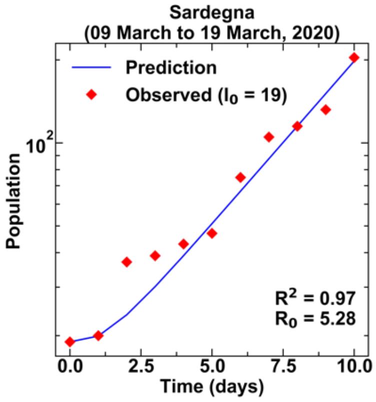

(17)

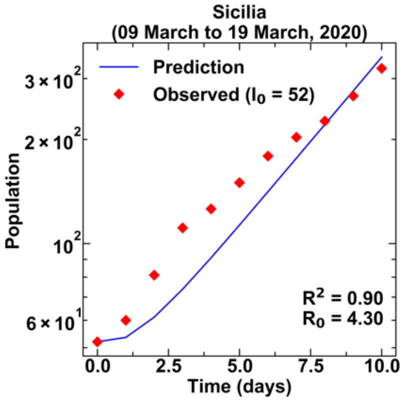

(18)

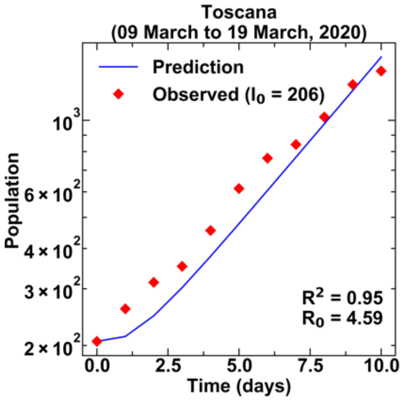

(19)

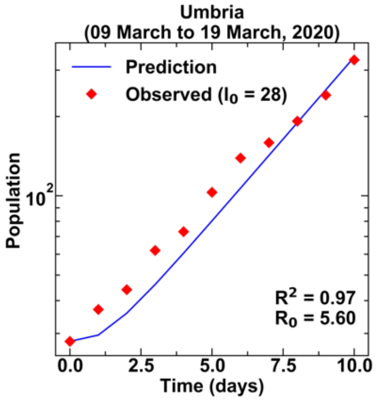

(20)

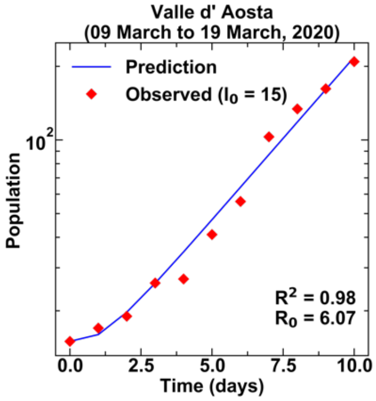

(21)

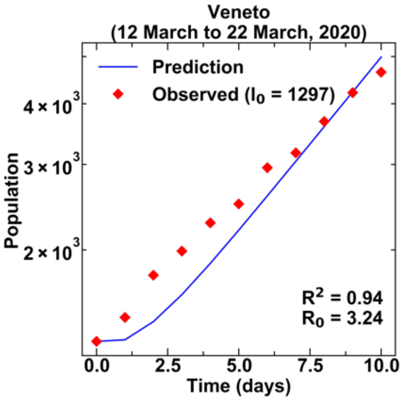

(22)

Fig. S3: SEIR model fitted against the observed data (from 23 March 2020 to 9 April 2020) for all the states of Italy.

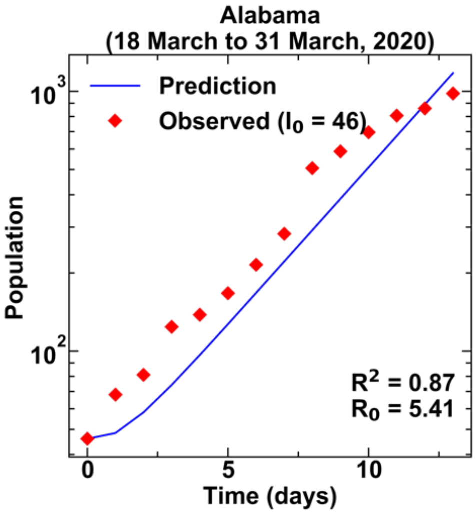
Alabama
(18 March to 31 March, 2020)
Prediction
Observed (I_0 = 46)
R^2 = 0.87
R_0 = 5.41
Population
Time (days)

(1)

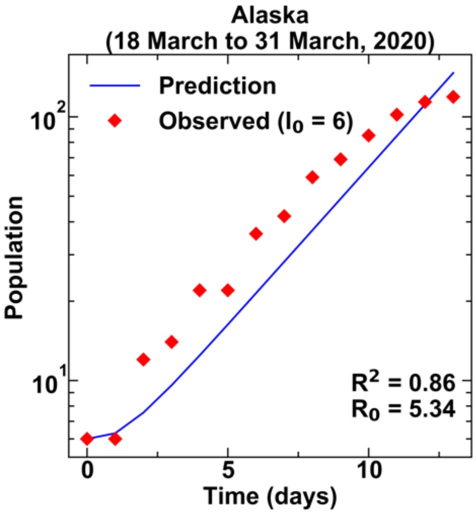
Alaska
(18 March to 31 March, 2020)
Prediction
Observed (I_0 = 6)
R^2 = 0.86
R_0 = 5.34
Population
Time (days)

(2)

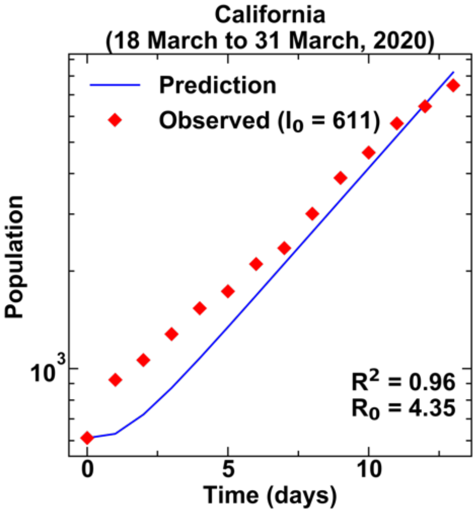
California
(18 March to 31 March, 2020)
Prediction
Observed (I_0 = 611)
R^2 = 0.96
R_0 = 4.35
Population
Time (days)

(3)

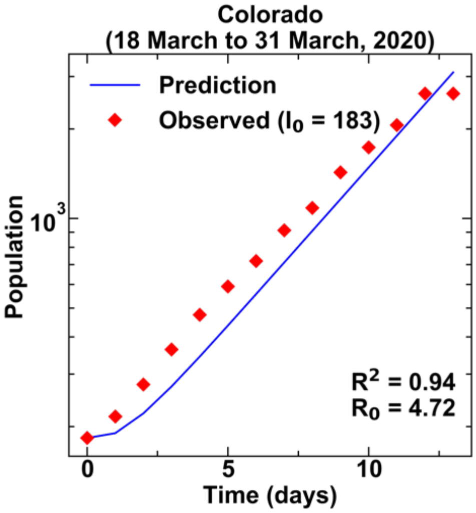
Colorado
(18 March to 31 March, 2020)
Prediction
Observed (I_0 = 183)
R^2 = 0.94
R_0 = 4.72
Population
Time (days)

(4)

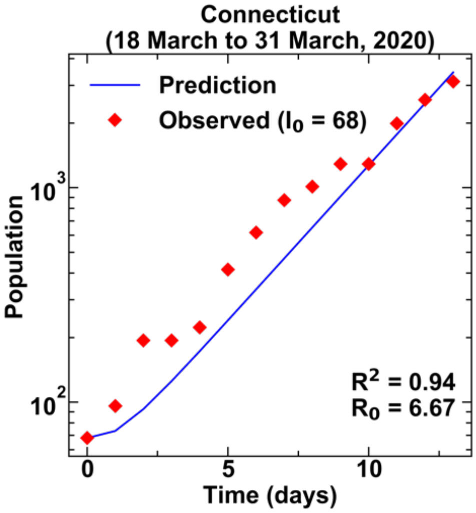
Connecticut
(18 March to 31 March, 2020)
Prediction
Observed (I_0 = 68)
R^2 = 0.94
R_0 = 6.67
Population
Time (days)

(5)

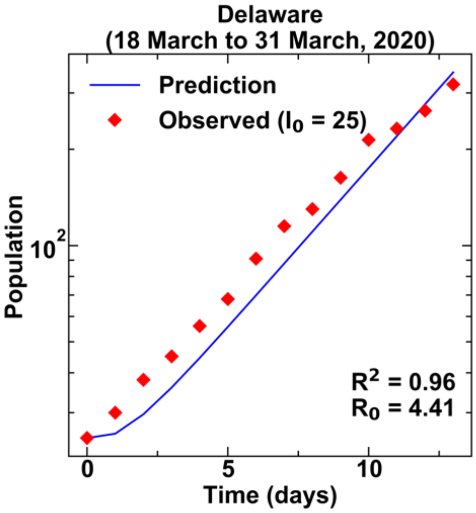
Delaware
(18 March to 31 March, 2020)
Prediction
Observed (I_0 = 25)
R^2 = 0.96
R_0 = 4.41
Population
Time (days)

(6)

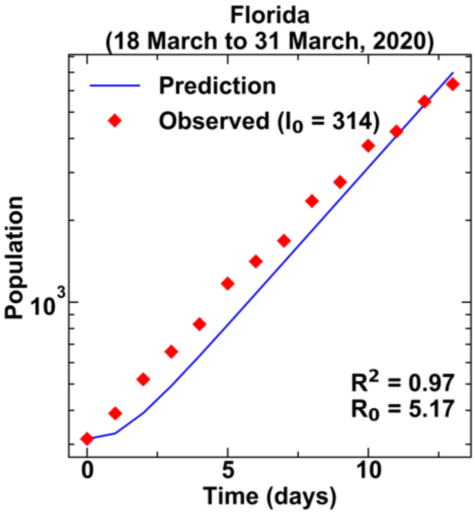
Florida
(18 March to 31 March, 2020)
Prediction
Observed (I_0 = 314)
R^2 = 0.97
R_0 = 5.17
Population
10^3
0
5
10
Time (days)

(7)

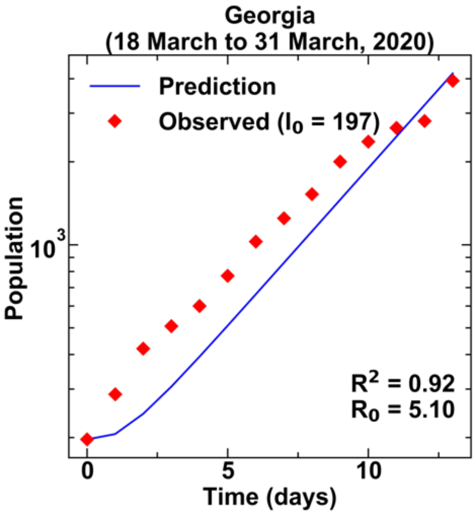
Georgia
(18 March to 31 March, 2020)
Prediction
Observed (I_0 = 197)
R^2 = 0.92
R_0 = 5.10
Population
10^3
0
5
10
Time (days)

(8)

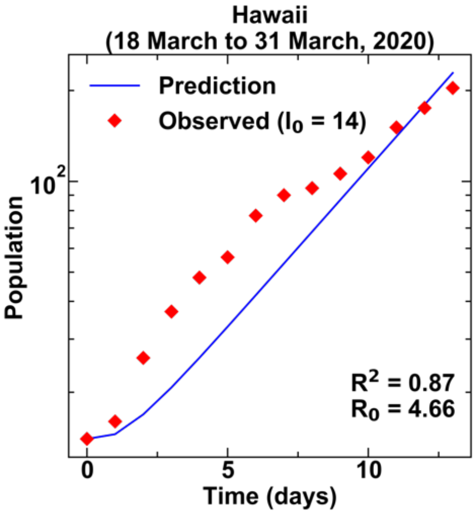
Hawaii
(18 March to 31 March, 2020)
Prediction
Observed (I_0 = 14)
R^2 = 0.87
R_0 = 4.66
Population
10^2
0
5
10
Time (days)

(9)

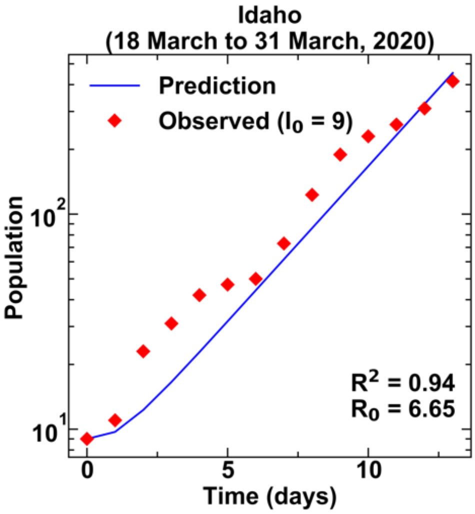
Idaho
(18 March to 31 March, 2020)
Prediction
Observed (I_0 = 9)
R^2 = 0.94
R_0 = 6.65
Population
10^2
10^1
0
5
10
Time (days)

(10)

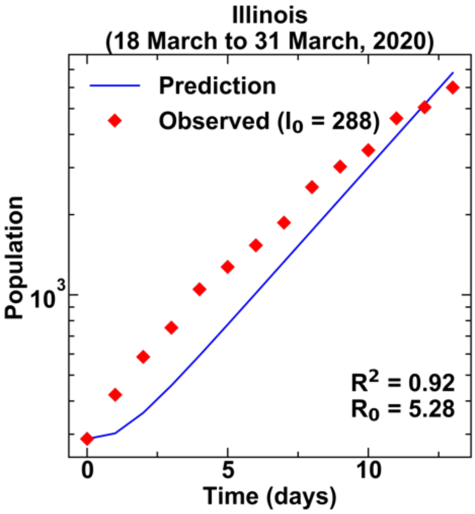
Illinois
(18 March to 31 March, 2020)
Prediction
Observed (I_0 = 288)
R^2 = 0.92
R_0 = 5.28
Population
10^3
0
5
10
Time (days)

(11)

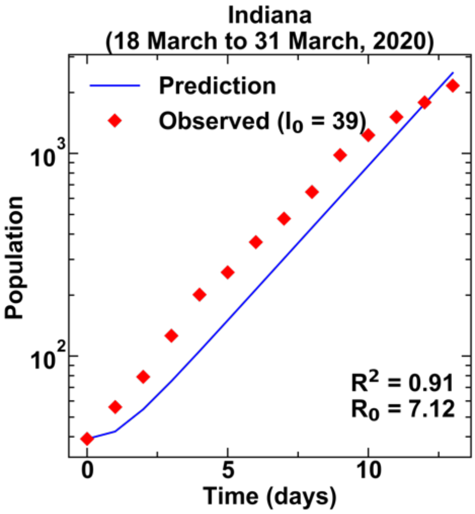
Indiana
(18 March to 31 March, 2020)
Prediction
Observed (I_0 = 39)
R^2 = 0.91
R_0 = 7.12
Population
10^3
10^2
0
5
10
Time (days)

(12)

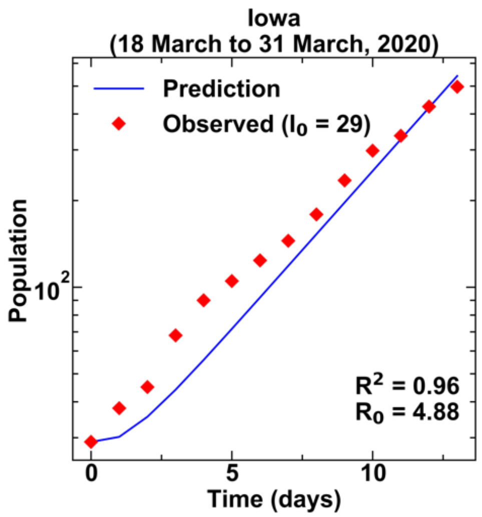

(13)

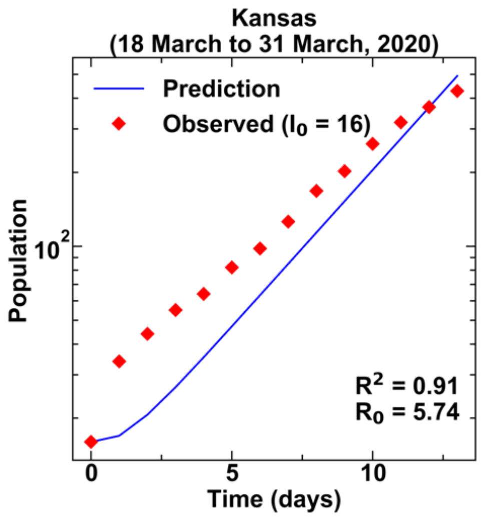

(14)

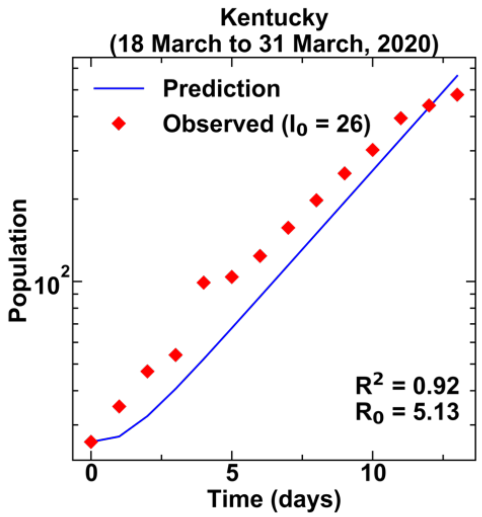

(15)

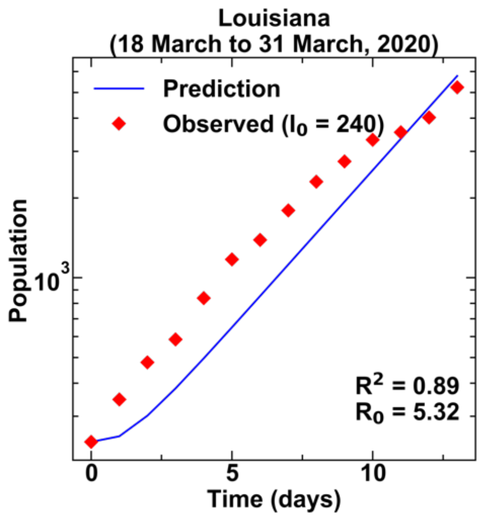

(16)

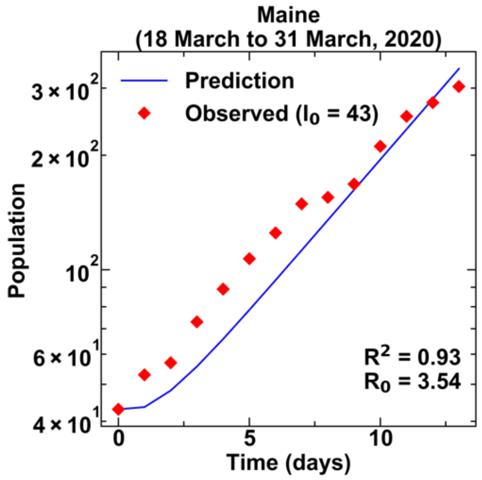

(17)

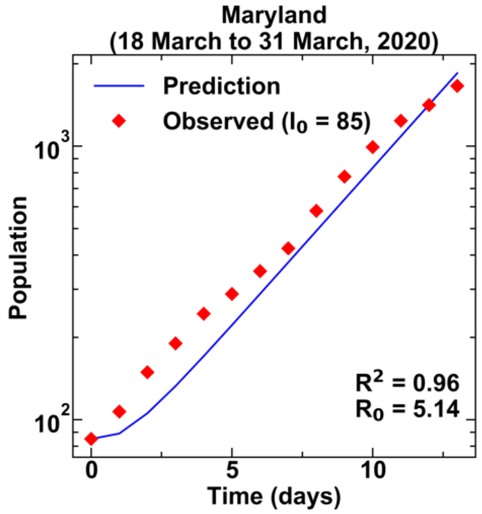

(18)

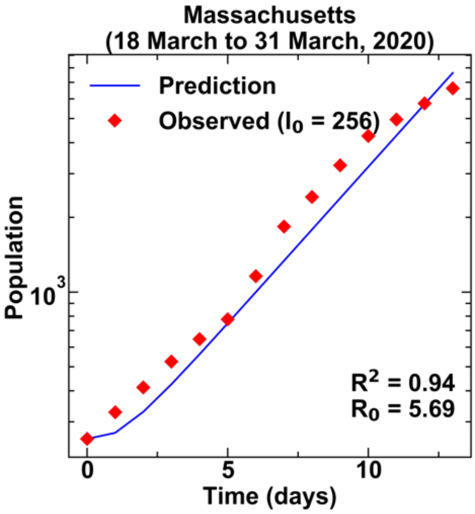

(19)

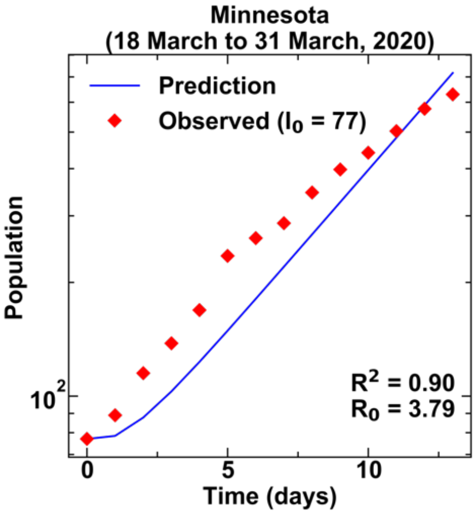

(20)

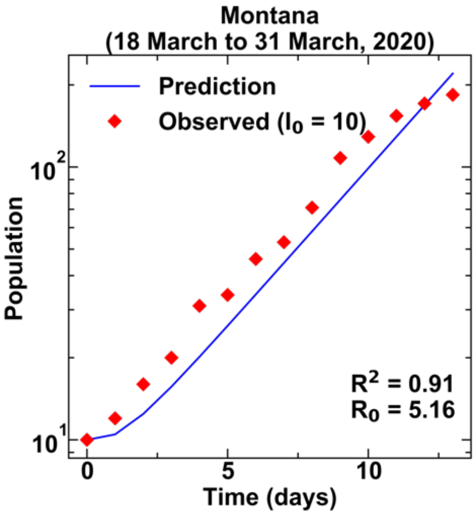

(21)

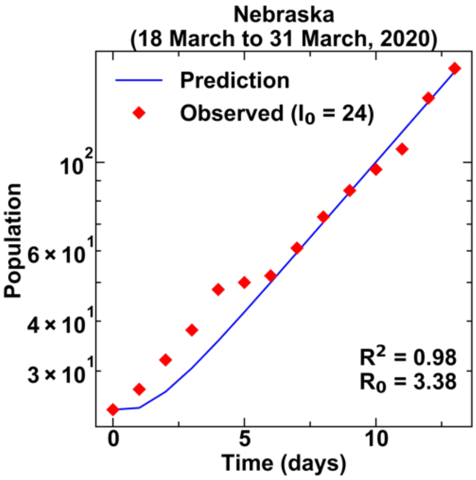

(22)

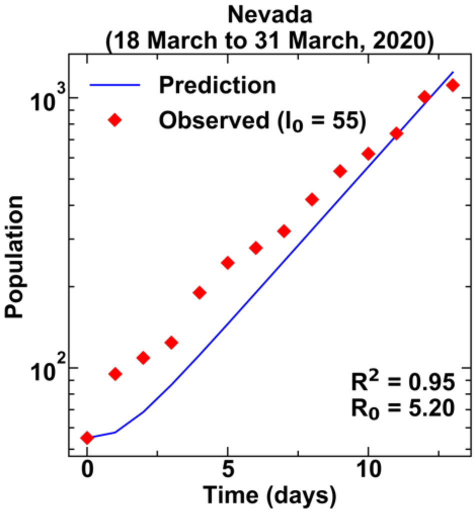

(23)

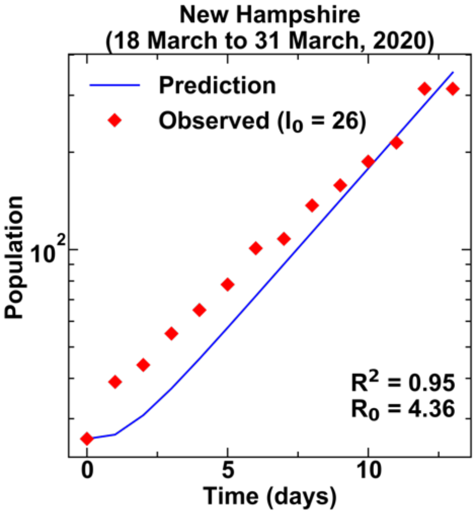

(24)

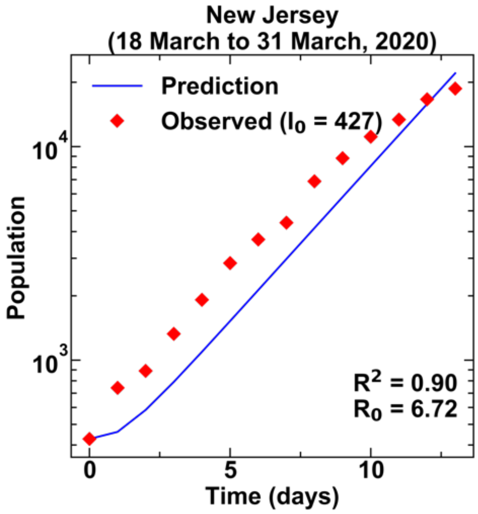

(25)

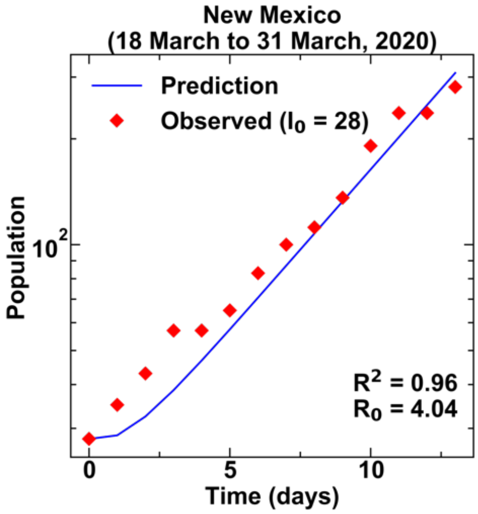

(26)

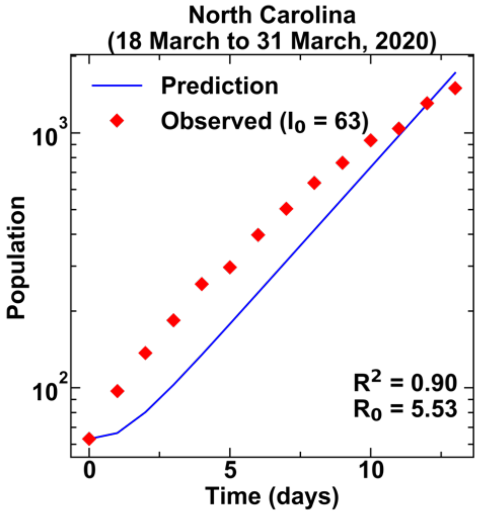

(27)

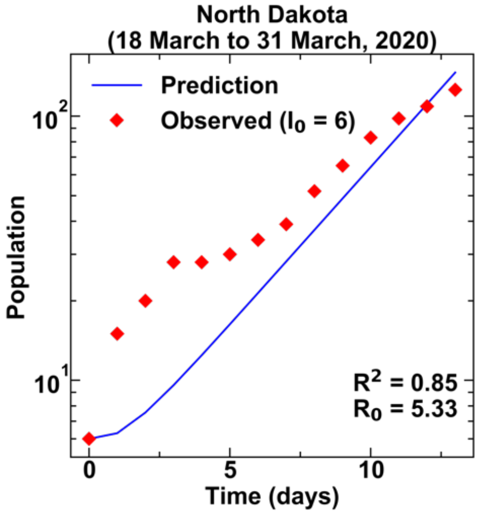

(28)

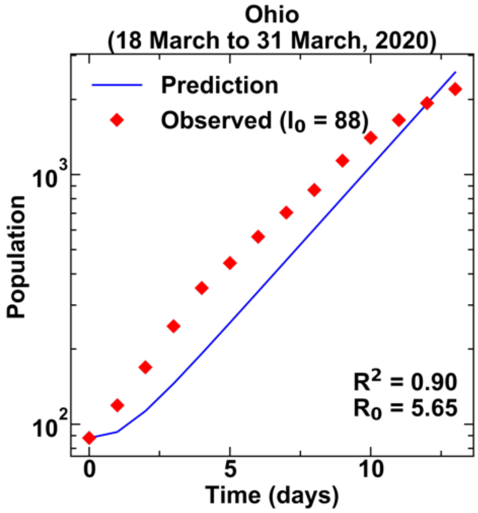

(29)

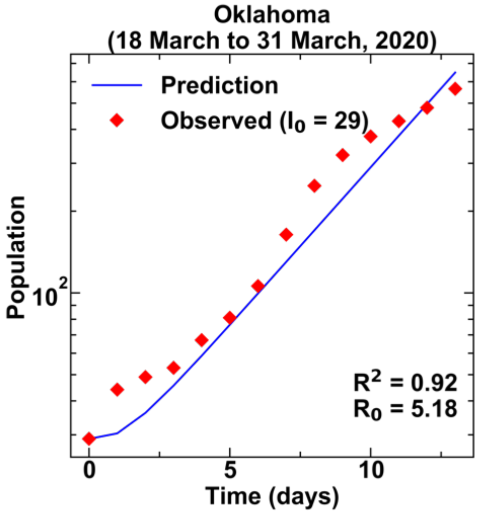

(30)

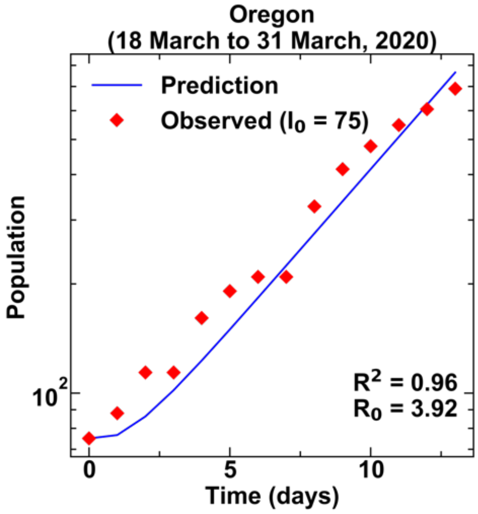

(31)

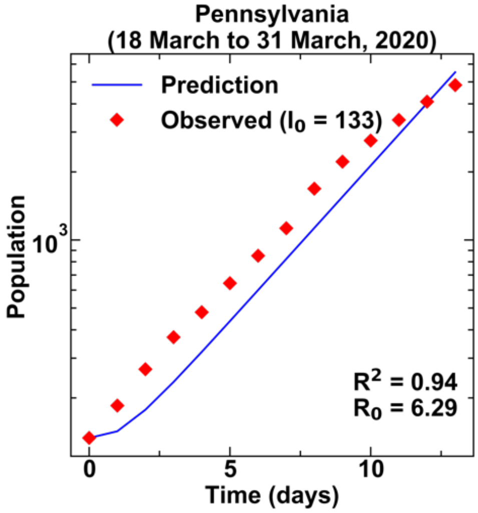

(32)

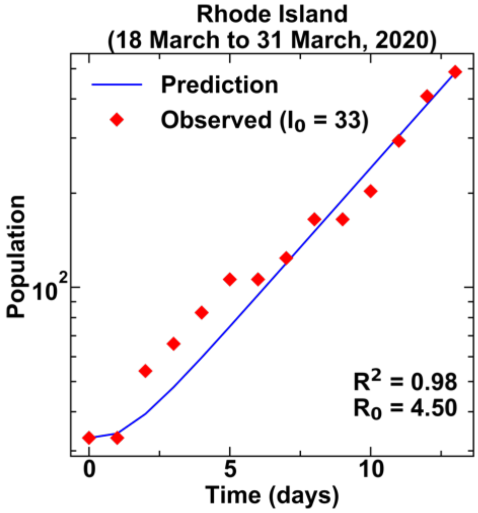

(33)

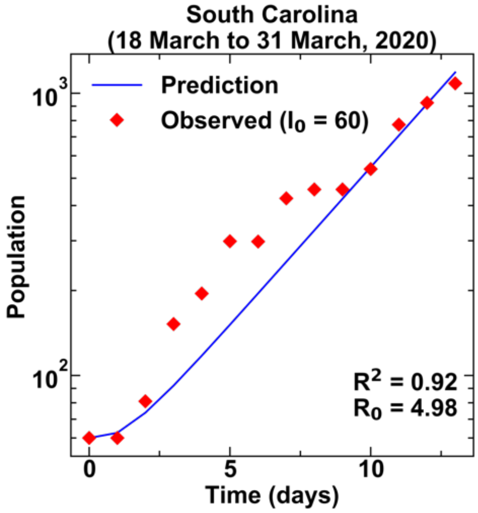

(34)

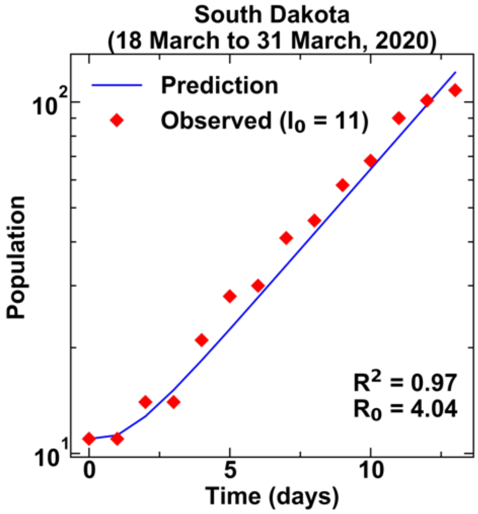

(35)

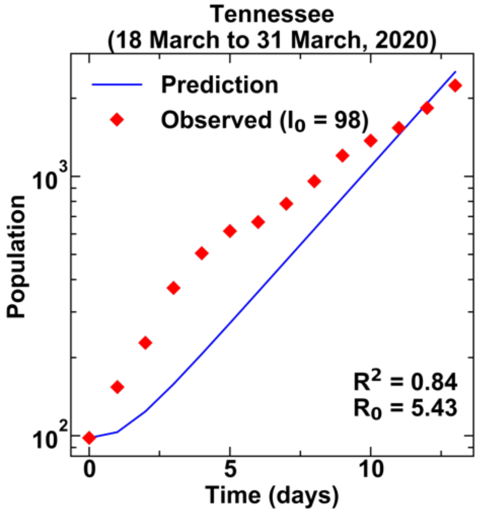

(36)

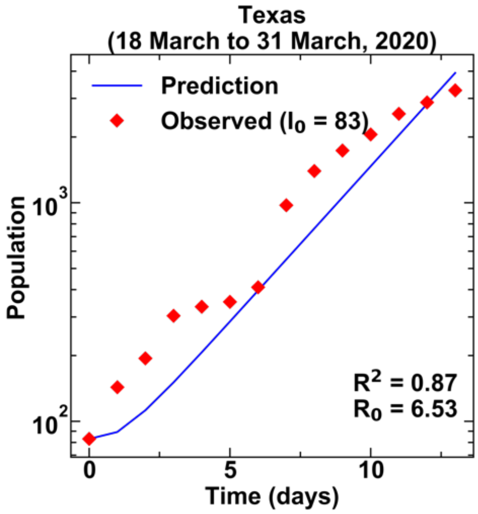

(37)

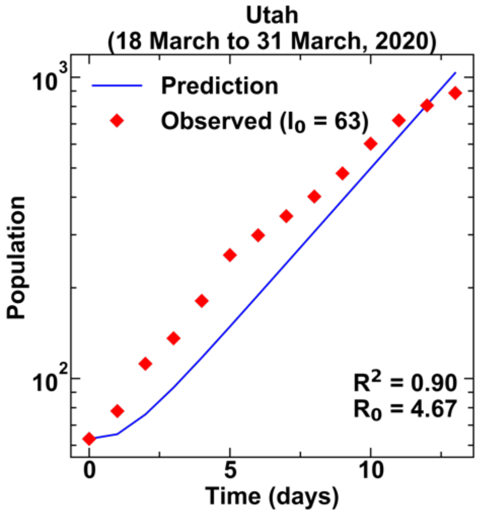

(38)

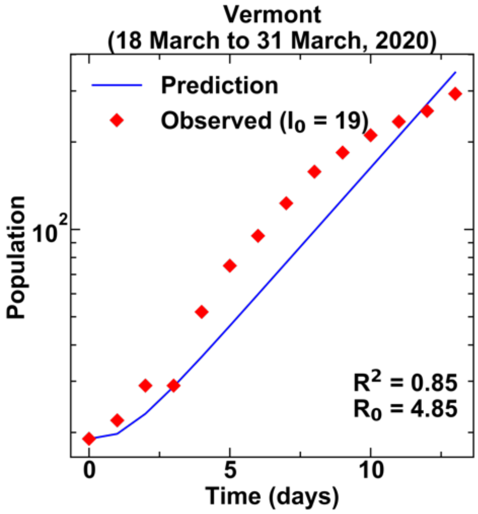

(39)

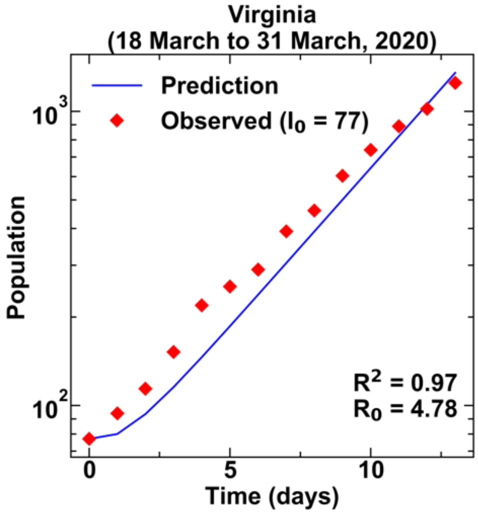

(40)

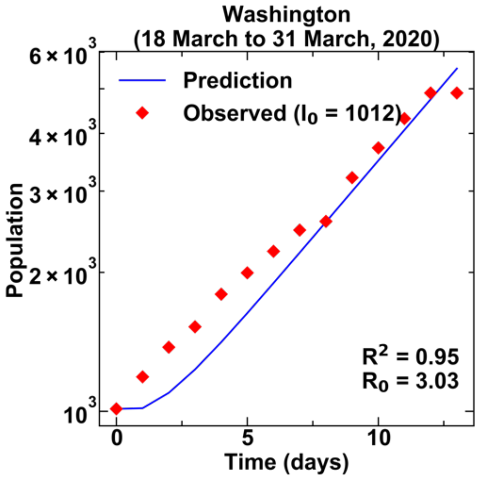

(41)

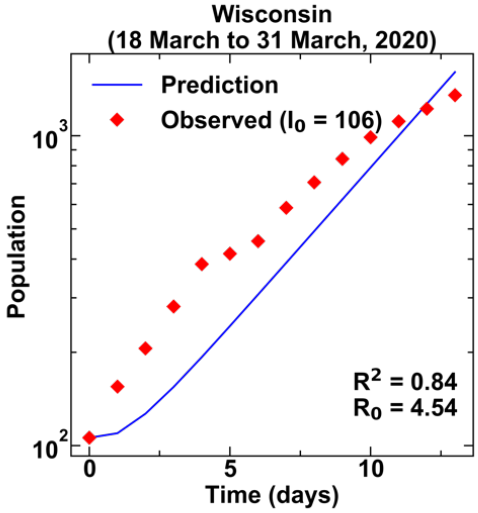

(42)

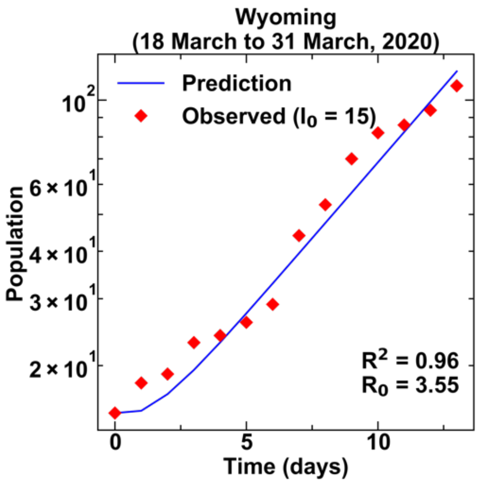

(43)

Fig. S4: SEIR model fitted against the observed data (from 4 March 2020 to 18 March 2020) for all the states of USA.

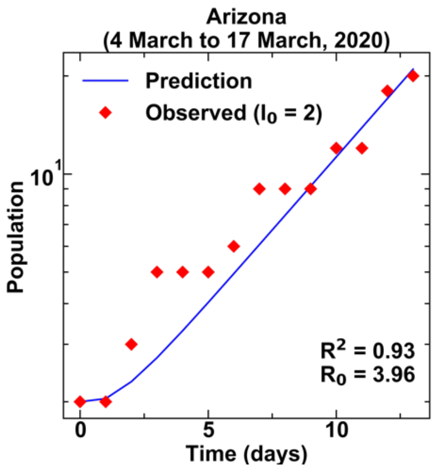
Arizona
(4 March to 17 March, 2020)
Prediction
Observed (I_0 = 2)
R^2 = 0.93
R_0 = 3.96
Population
Time (days)

(1)

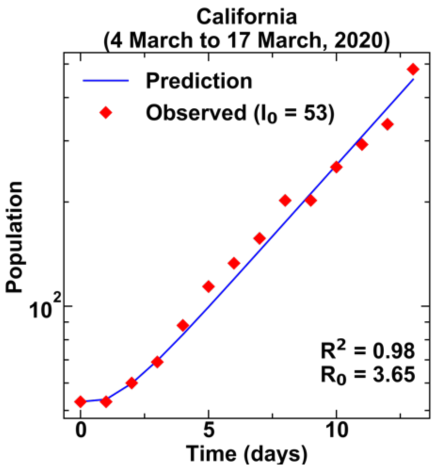
California
(4 March to 17 March, 2020)
Prediction
Observed (I_0 = 53)
R^2 = 0.98
R_0 = 3.65
Population
Time (days)

(2)

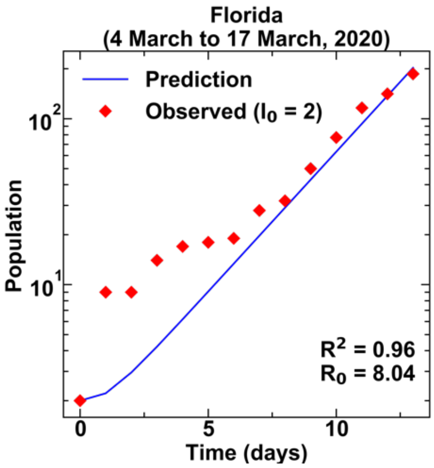
Florida
(4 March to 17 March, 2020)
Prediction
Observed (I_0 = 2)
R^2 = 0.96
R_0 = 8.04
Population
Time (days)

(3)

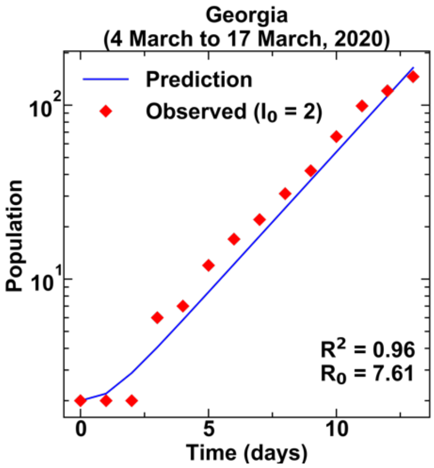
Georgia
(4 March to 17 March, 2020)
Prediction
Observed (I_0 = 2)
R^2 = 0.96
R_0 = 7.61
Population
Time (days)

(4)

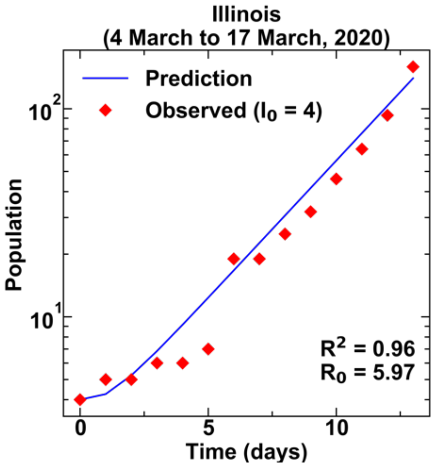
Illinois
(4 March to 17 March, 2020)
Prediction
Observed (I_0 = 4)
R^2 = 0.96
R_0 = 5.97
Population
Time (days)

(5)

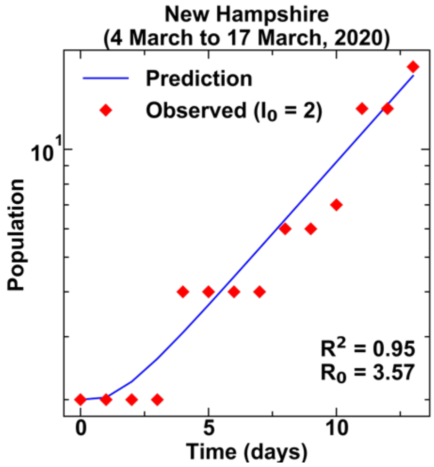
New Hampshire
(4 March to 17 March, 2020)
Prediction
Observed (I_0 = 2)
R^2 = 0.95
R_0 = 3.57
Population
Time (days)

(6)

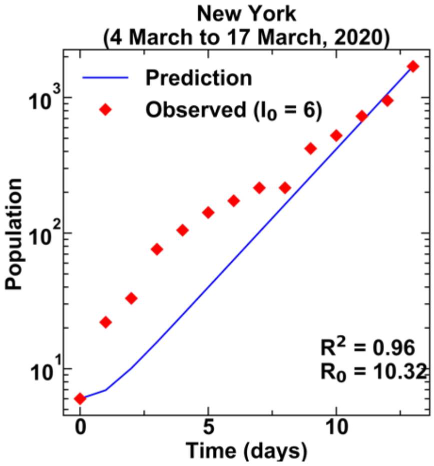

(7)

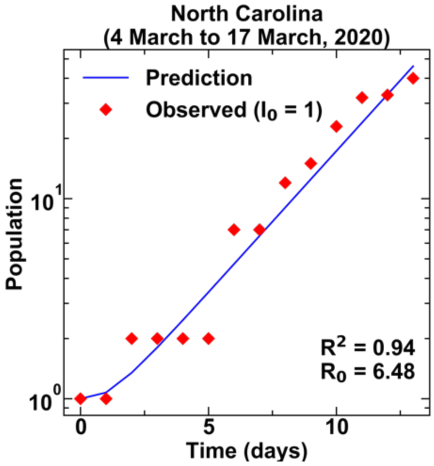

(8)

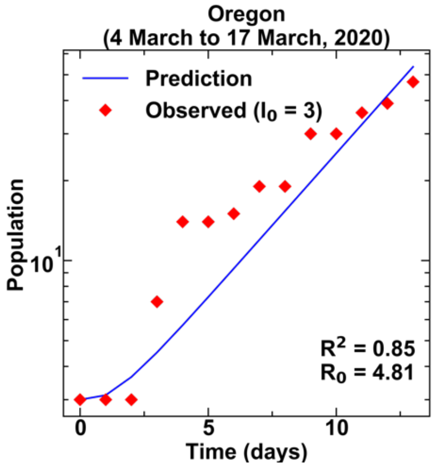

(9)

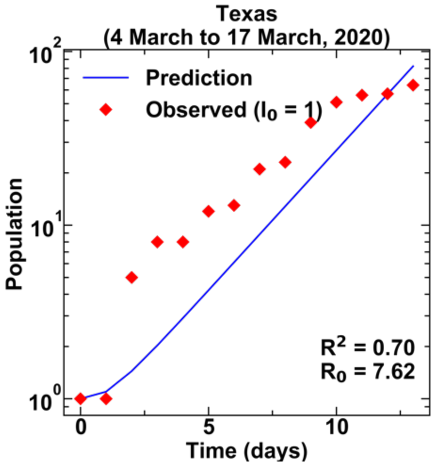

(10)

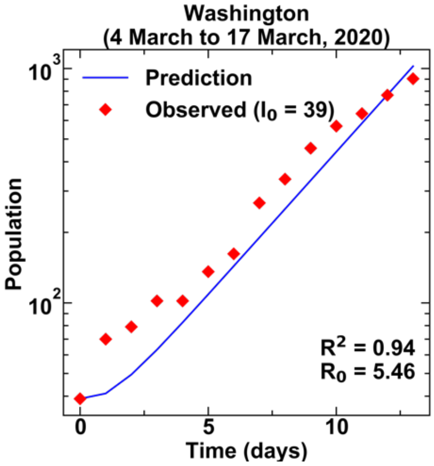

(11)

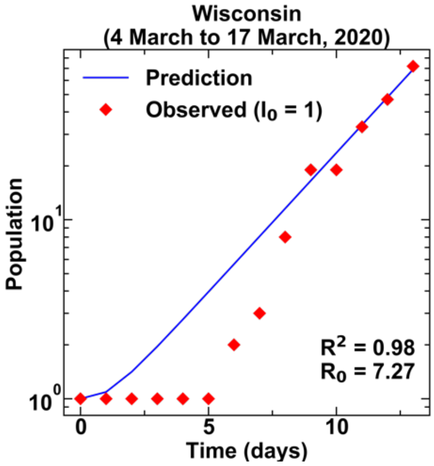

(12)

Fig. S5: SEIR model fitted against the observed data (from 18 March 2020 to 9 April 2020) for all the states of USA.

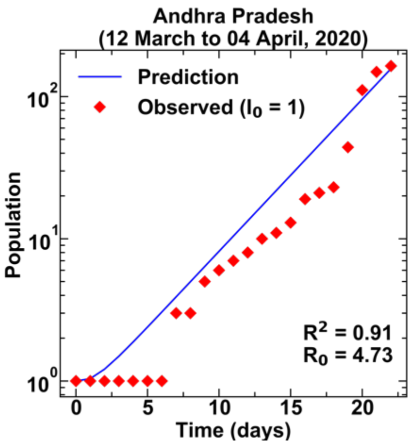
Andhra Pradesh
(12 March to 04 April, 2020)
Prediction
Observed (I_0 = 1)
R^2 = 0.91
R_0 = 4.73
Population
Time (days)

(1)

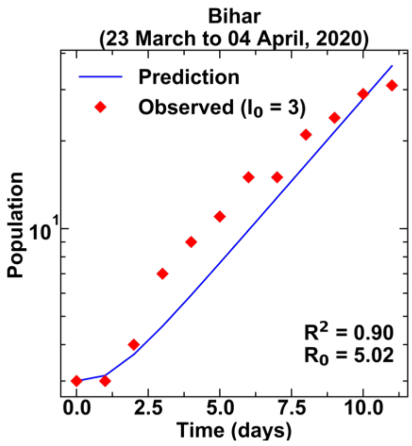
Bihar
(23 March to 04 April, 2020)
Prediction
Observed (I_0 = 3)
R^2 = 0.90
R_0 = 5.02
Population
Time (days)

(2)

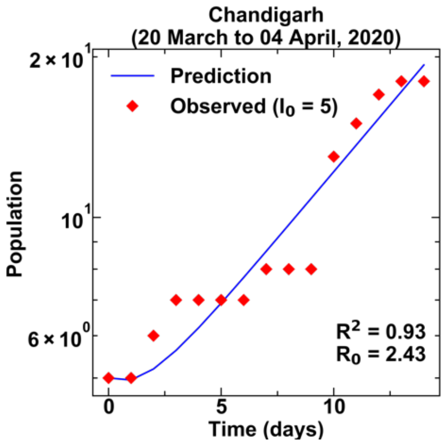
Chandigarh
(20 March to 04 April, 2020)
Prediction
Observed (I_0 = 5)
R^2 = 0.93
R_0 = 2.43
Population
Time (days)

(3)

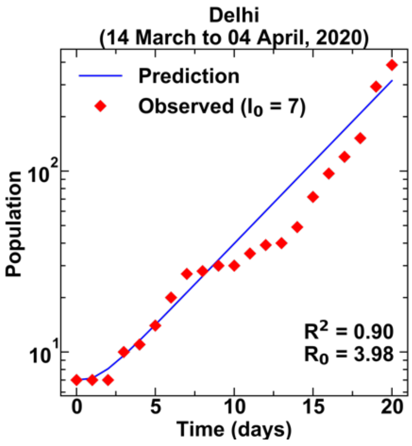
Delhi
(14 March to 04 April, 2020)
Prediction
Observed (I_0 = 7)
R^2 = 0.90
R_0 = 3.98
Population
Time (days)

(4)

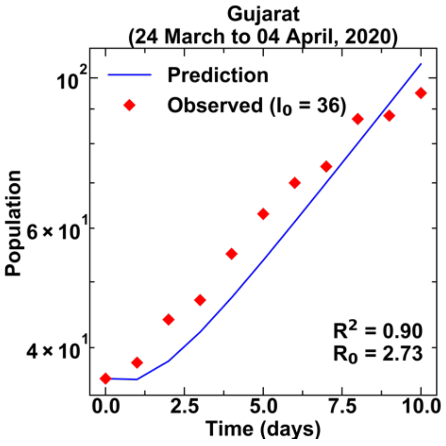
Gujarat
(24 March to 04 April, 2020)
Prediction
Observed (I_0 = 36)
R^2 = 0.90
R_0 = 2.73
Population
Time (days)

(5)

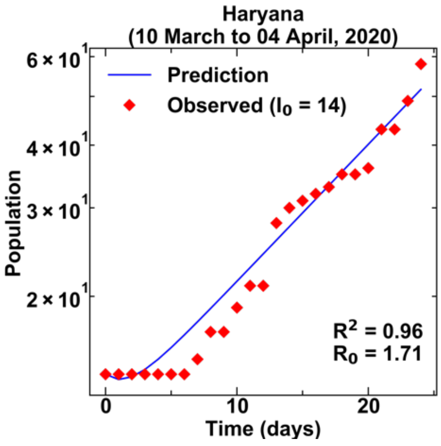
Haryana
(10 March to 04 April, 2020)
Prediction
Observed (I_0 = 14)
R^2 = 0.96
R_0 = 1.71
Population
Time (days)

(6)

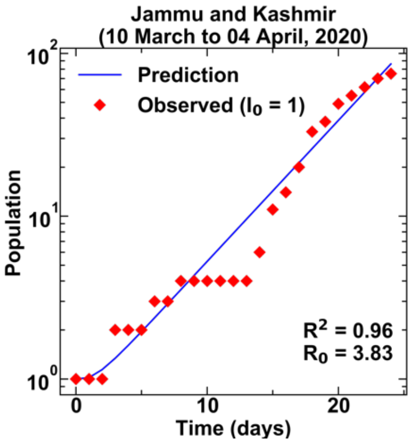
Jammu and Kashmir
(10 March to 04 April, 2020)
Prediction
Observed (I_0 = 1)
R^2 = 0.96
R_0 = 3.83
Population
Time (days)

(7)

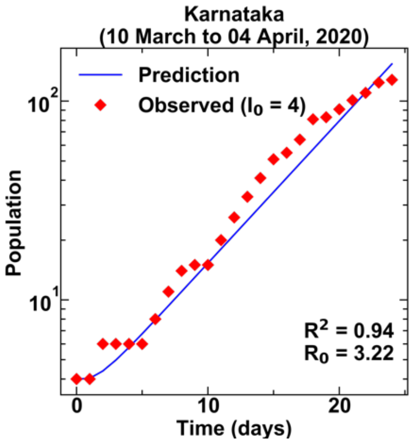
Karnataka
(10 March to 04 April, 2020)
Prediction
Observed (I_0 = 4)
R^2 = 0.94
R_0 = 3.22
Population
Time (days)

(8)

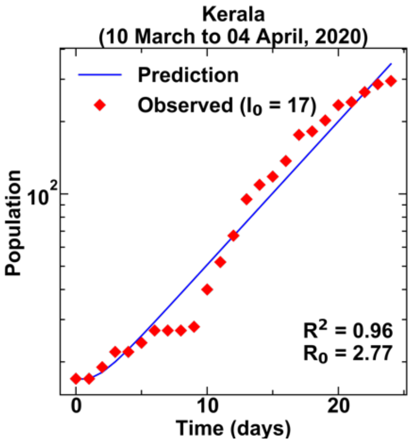
Kerala
(10 March to 04 April, 2020)
Prediction
Observed (I_0 = 17)
R^2 = 0.96
R_0 = 2.77
Population
Time (days)

(9)

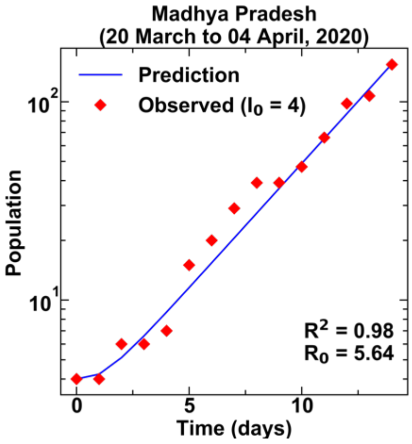
Madhya Pradesh
(20 March to 04 April, 2020)
Prediction
Observed (I_0 = 4)
R^2 = 0.98
R_0 = 5.64
Population
Time (days)

(10)

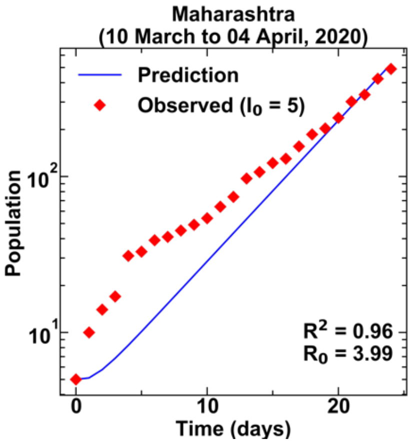
Maharashtra
(10 March to 04 April, 2020)
Prediction
Observed (I_0 = 5)
R^2 = 0.96
R_0 = 3.99
Population
Time (days)

(11)

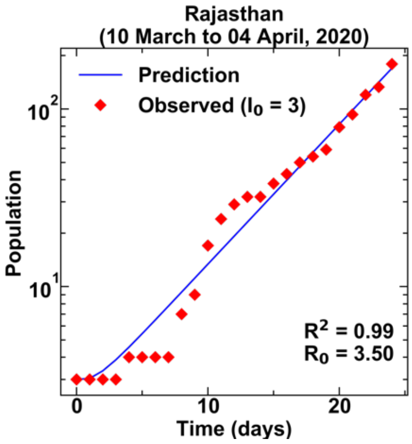
Rajasthan
(10 March to 04 April, 2020)
Prediction
Observed (I_0 = 3)
R^2 = 0.99
R_0 = 3.50
Population
Time (days)

(12)

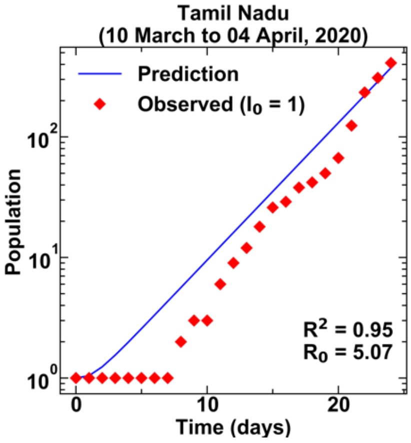

(13)

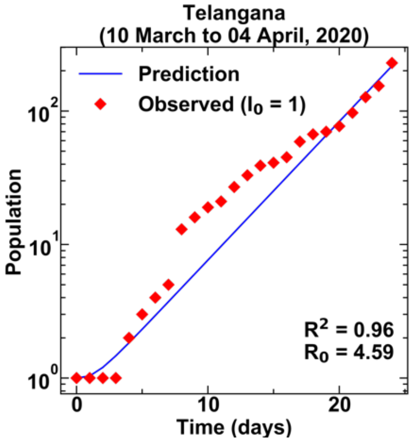

(14)

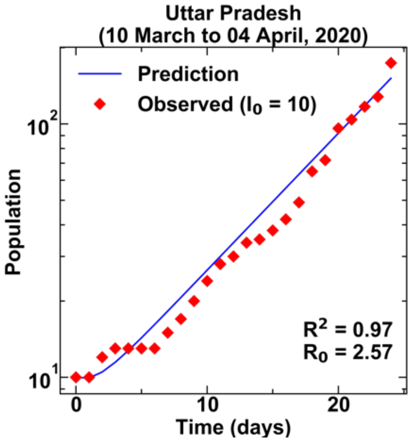

(15)

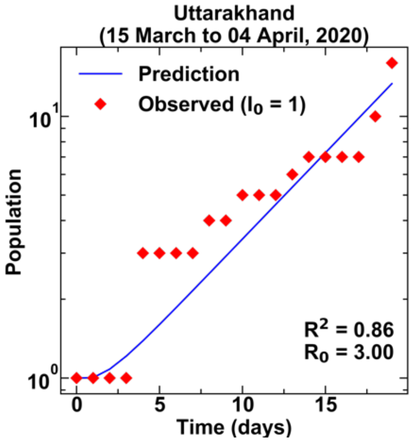

(16)

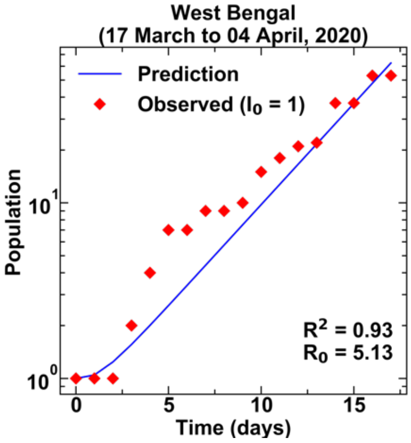

(17)

Fig. S6: SEIR model fitted against the observed data (from 10 March 2020 to 4 April 2020) for all the states of India.

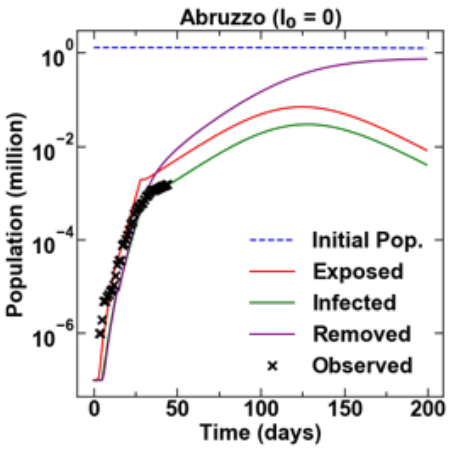
Abruzzo (I_0 = 0)
Population (million)
Time (days)
Initial Pop.
Exposed
Infected
Removed
Observed

(1)

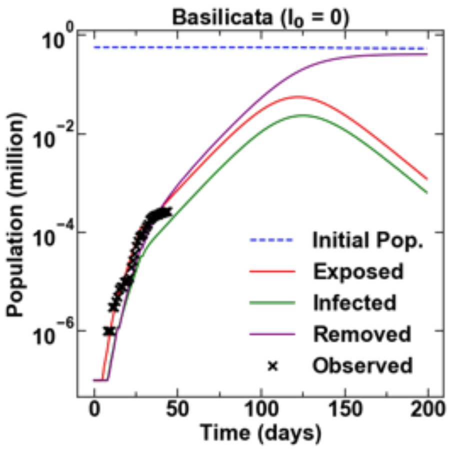
Basilicata (I_0 = 0)
Population (million)
Time (days)
Initial Pop.
Exposed
Infected
Removed
Observed

(2)

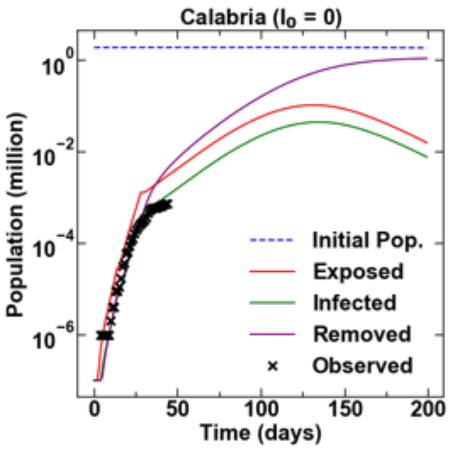
Calabria (I_0 = 0)
Population (million)
Time (days)
Initial Pop.
Exposed
Infected
Removed
Observed

(3)

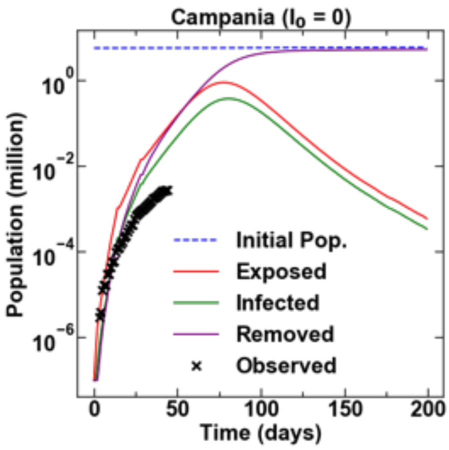
Campania (I_0 = 0)
Population (million)
Time (days)
Initial Pop.
Exposed
Infected
Removed
Observed

(4)

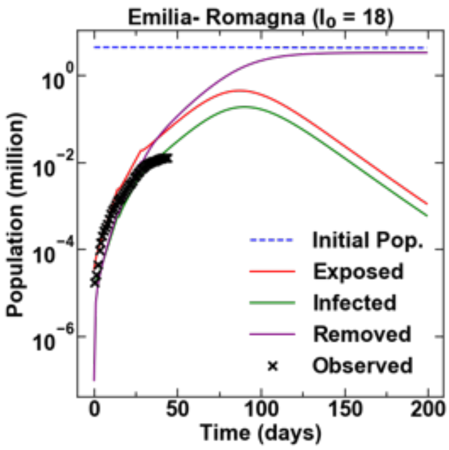
Emilia- Romagna (I_0 = 18)
Population (million)
Time (days)
Initial Pop.
Exposed
Infected
Removed
Observed

(5)

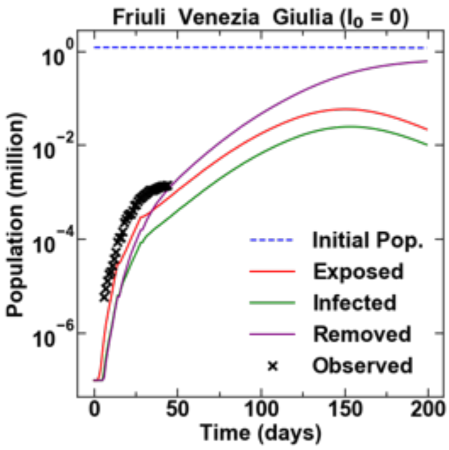
Friuli Venezia Giulia (I_0 = 0)
Population (million)
Time (days)
Initial Pop.
Exposed
Infected
Removed
Observed

(6)

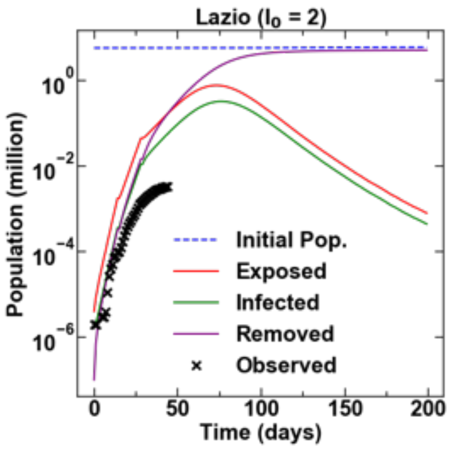
Lazio (I_0 = 2)
Population (million)
Time (days)
Initial Pop.
Exposed
Infected
Removed
Observed

(7)

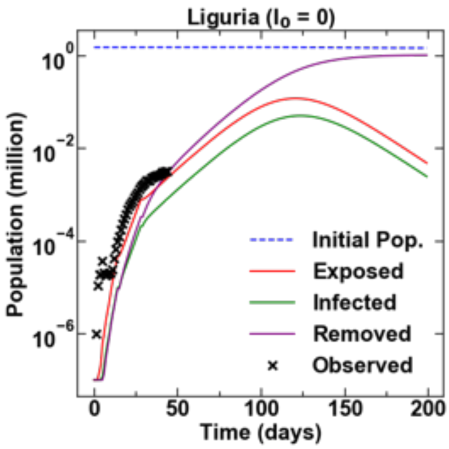
Liguria (I_0 = 0)
Population (million)
Time (days)
Initial Pop.
Exposed
Infected
Removed
Observed

(8)

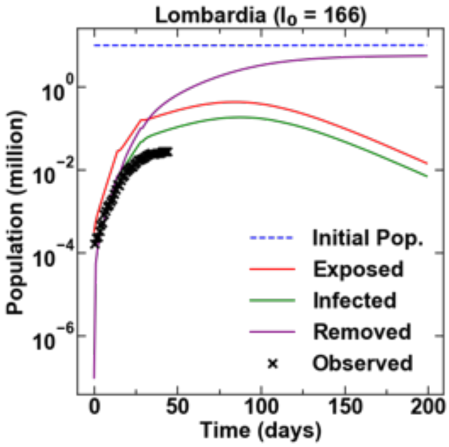
Lombardia (I_0 = 166)
Population (million)
Time (days)
Initial Pop.
Exposed
Infected
Removed
Observed

(9)

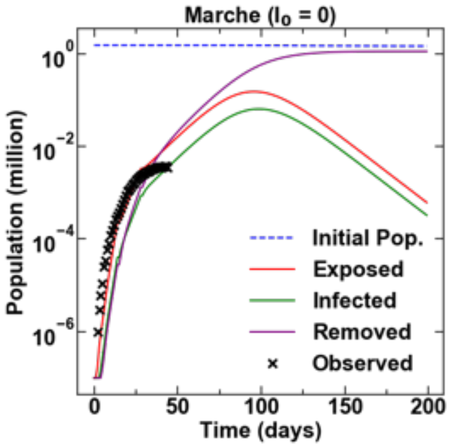
Marche (I_0 = 0)
Population (million)
Time (days)
Initial Pop.
Exposed
Infected
Removed
Observed

(10)

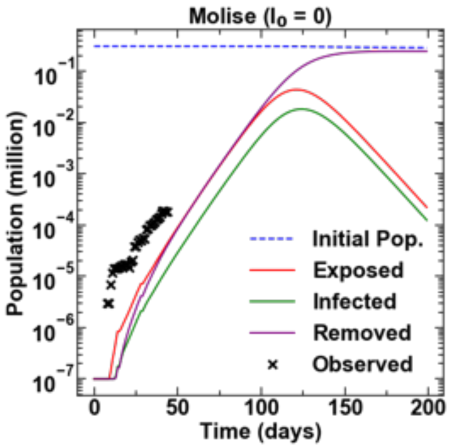
Molise (I_0 = 0)
Population (million)
Time (days)
Initial Pop.
Exposed
Infected
Removed
Observed

(11)

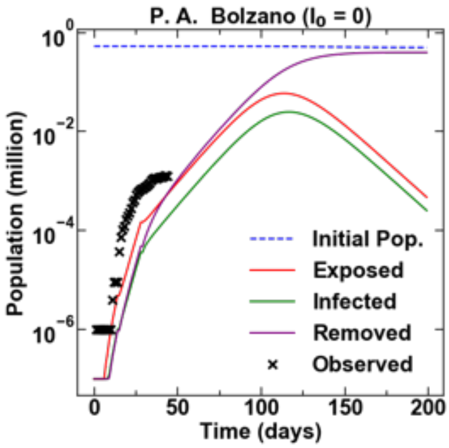
P. A. Bolzano (I_0 = 0)
Population (million)
Time (days)
Initial Pop.
Exposed
Infected
Removed
Observed

(12)

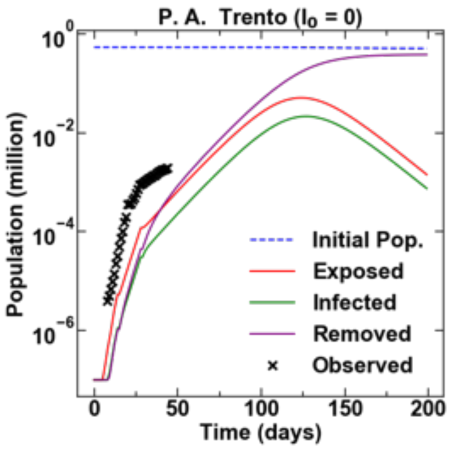

(13)

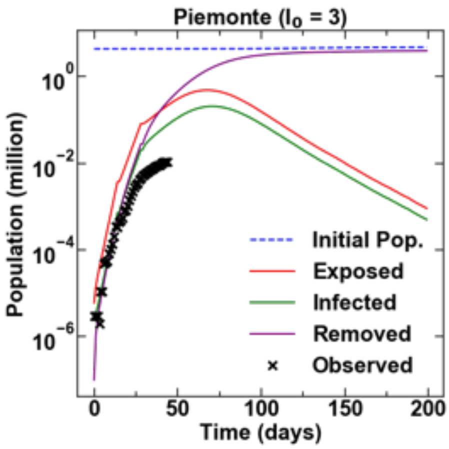

(14)

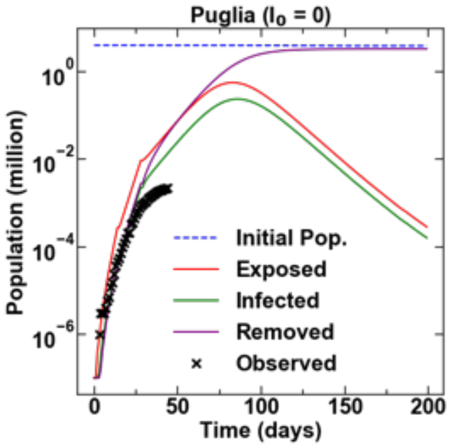

(15)

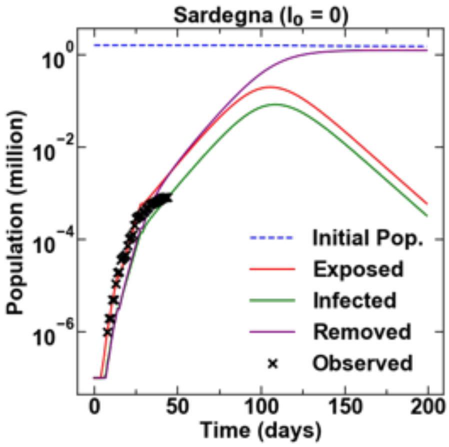

(16)

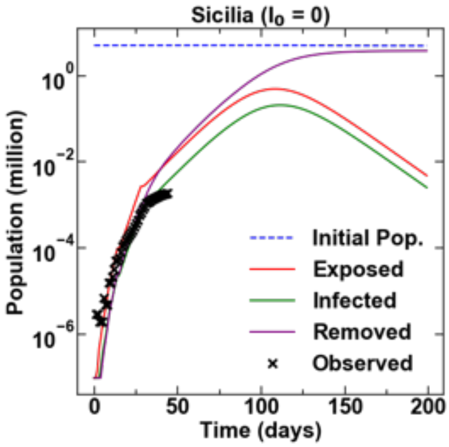

(17)

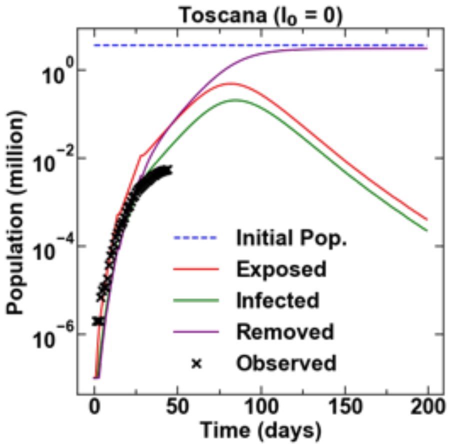

(18)

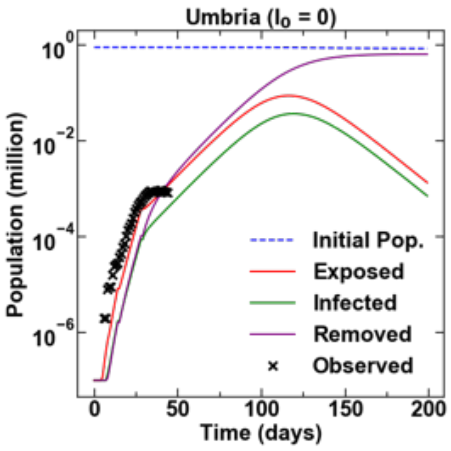

(19)

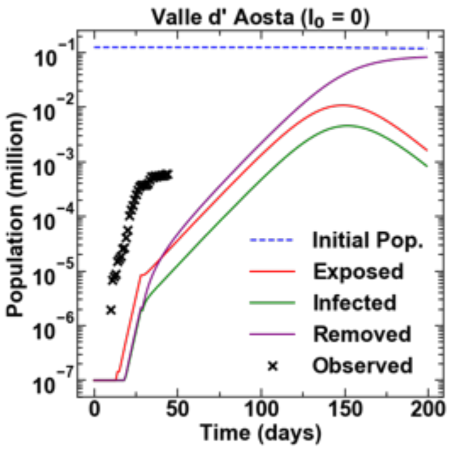

(20)

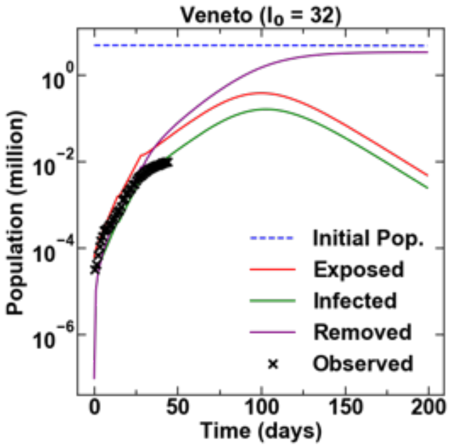

(21)

Fig. S7: Mapping of the pandemic in all the states of Italy as predicted by AICSEIR model in comparison to the observed data.

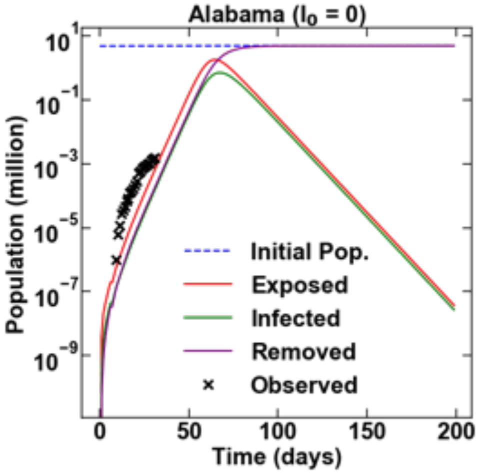
Alabama (I_0 = 0)
Population (million)
Time (days)
Initial Pop.
Exposed
Infected
Removed
Observed

(1)

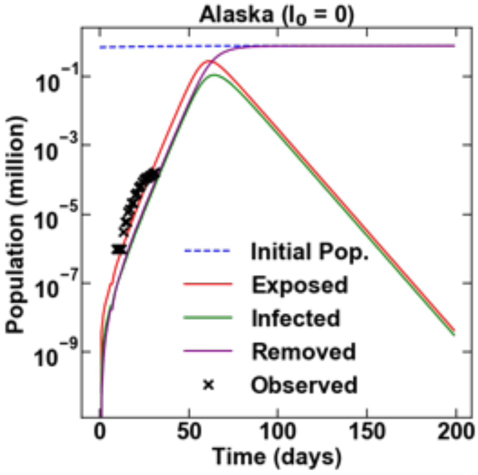
Alaska (I_0 = 0)
Population (million)
Time (days)
Initial Pop.
Exposed
Infected
Removed
Observed

(2)

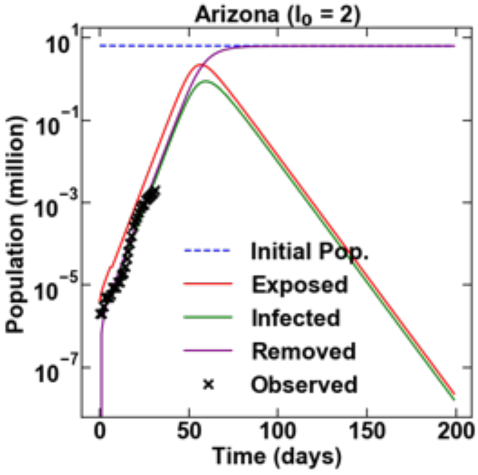
Arizona (I_0 = 2)
Population (million)
Time (days)
Initial Pop.
Exposed
Infected
Removed
Observed

(3)

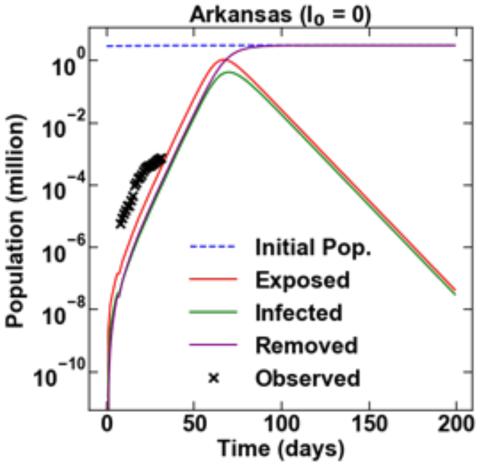
Arkansas (I_0 = 0)
Population (million)
Time (days)
Initial Pop.
Exposed
Infected
Removed
Observed

(4)

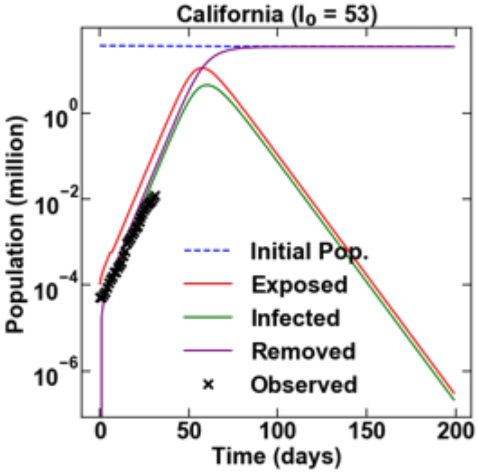
California (I_0 = 53)
Population (million)
Time (days)
Initial Pop.
Exposed
Infected
Removed
Observed

(5)

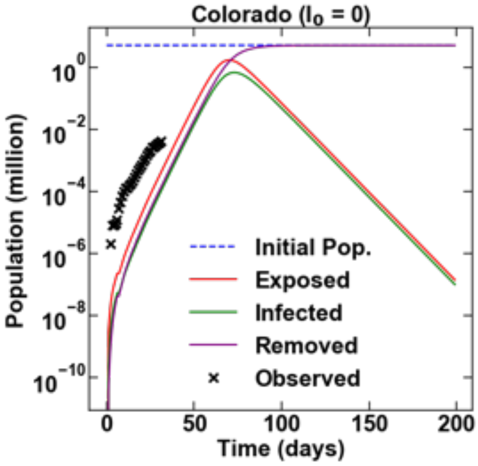
Colorado (I_0 = 0)
Population (million)
Time (days)
Initial Pop.
Exposed
Infected
Removed
Observed

(6)

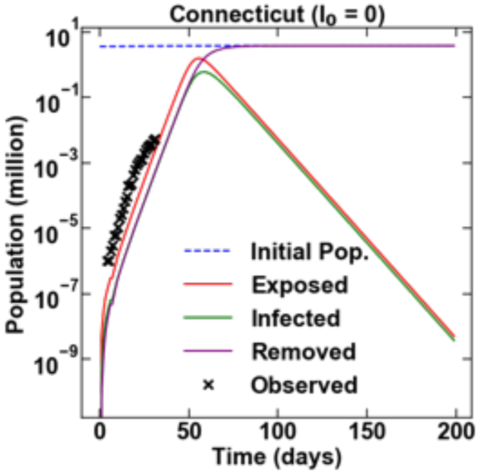
Connecticut (I_0 = 0)
Population (million)
Time (days)
Initial Pop.
Exposed
Infected
Removed
Observed

(7)

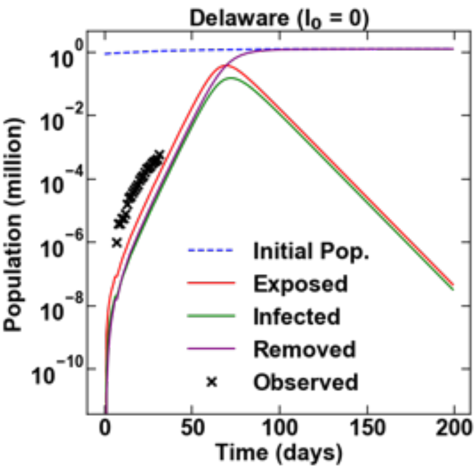
Delaware (I_0 = 0)
Population (million)
Time (days)
Initial Pop.
Exposed
Infected
Removed
Observed

(8)

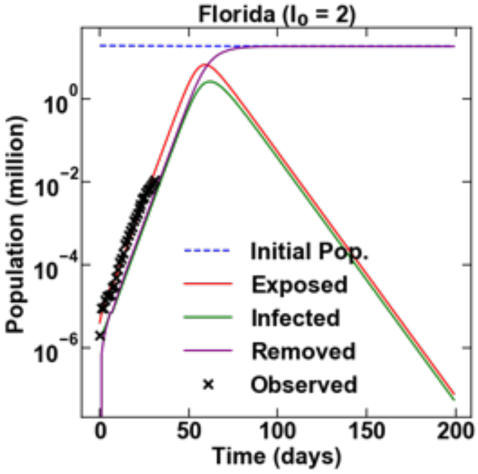
Florida (I_0 = 2)
Population (million)
Time (days)
Initial Pop.
Exposed
Infected
Removed
Observed

(9)

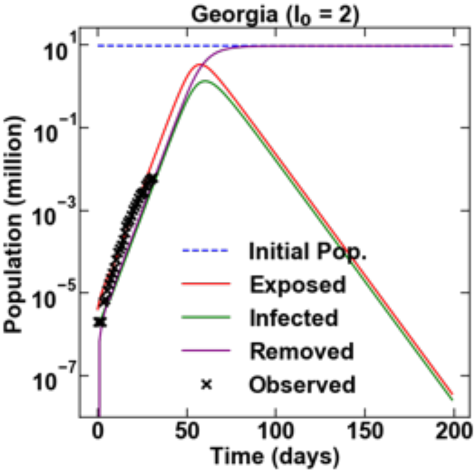
Georgia (I_0 = 2)
Population (million)
Time (days)
Initial Pop.
Exposed
Infected
Removed
Observed

(10)

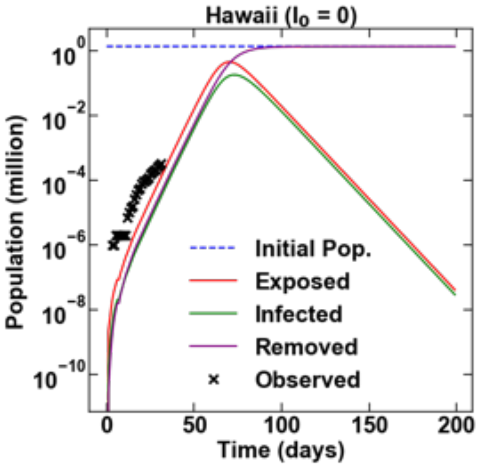
Hawaii (I_0 = 0)
Population (million)
Time (days)
Initial Pop.
Exposed
Infected
Removed
Observed

(11)

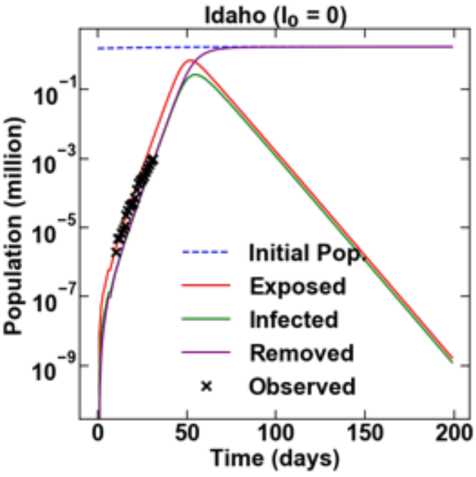
Idaho (I_0 = 0)
Population (million)
Time (days)
Initial Pop.
Exposed
Infected
Removed
Observed

(12)

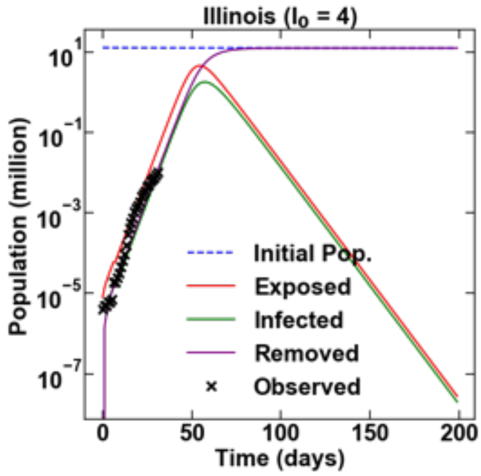

(13)

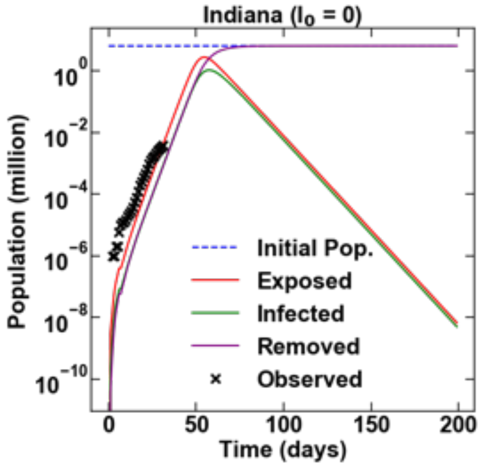

(14)

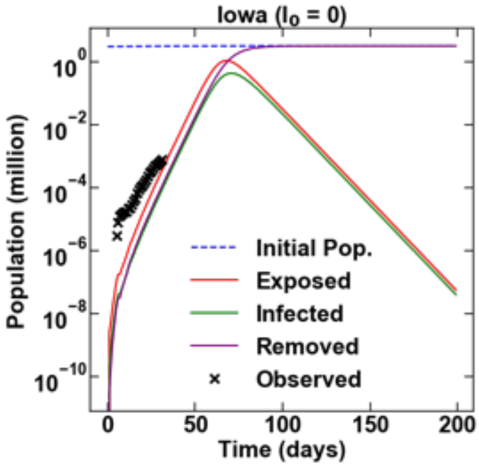

(15)

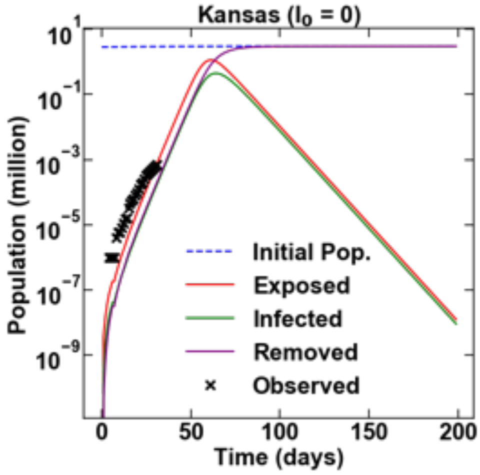

(16)

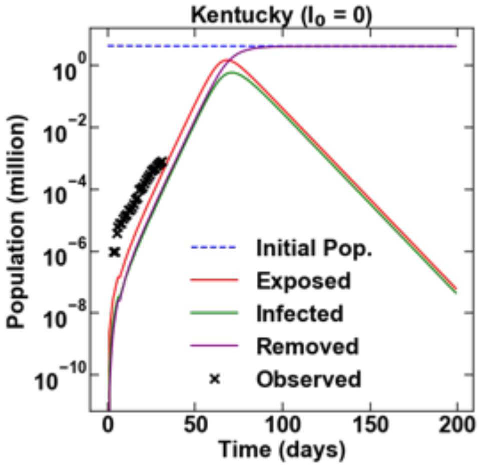

(17)

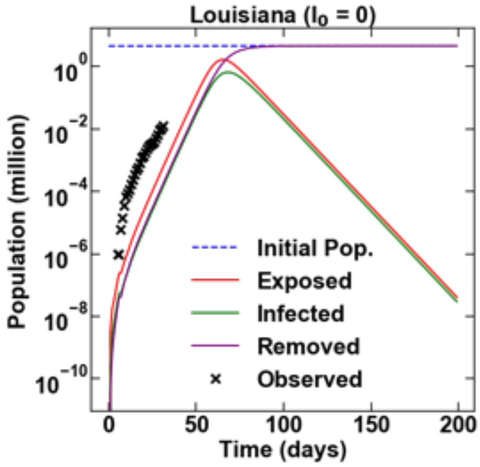

(18)

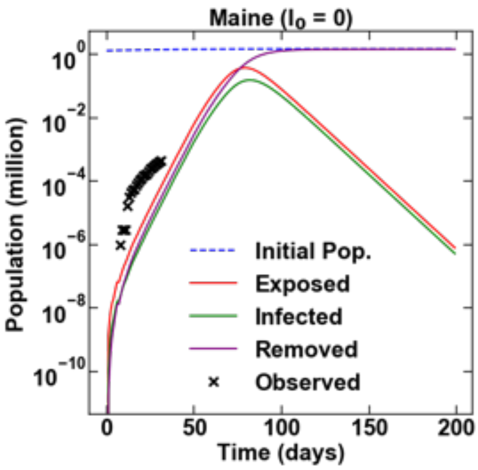

(19)

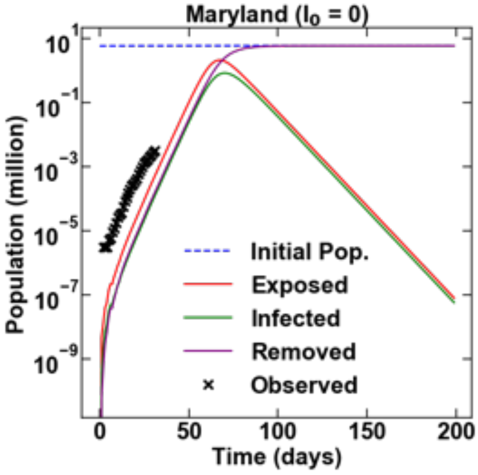

(20)

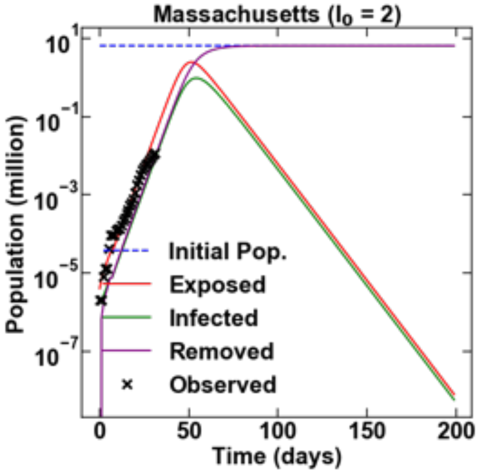

(21)

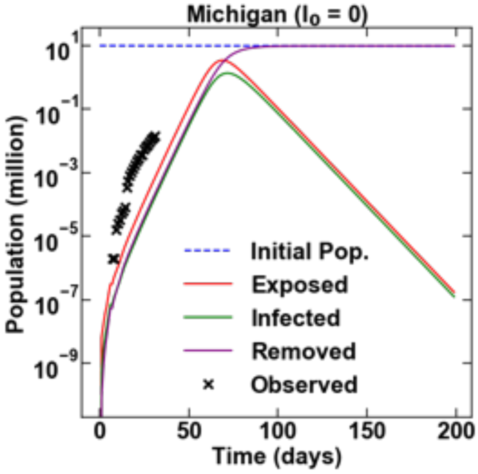

(22)

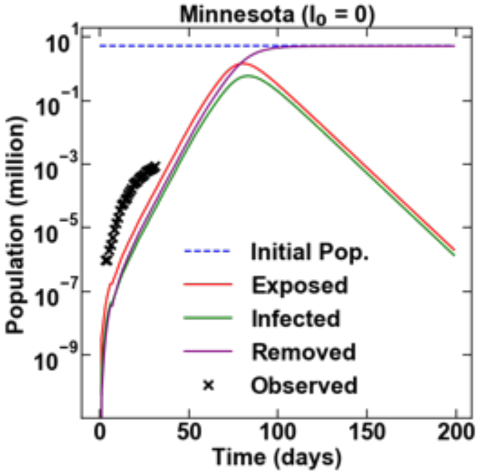

(23)

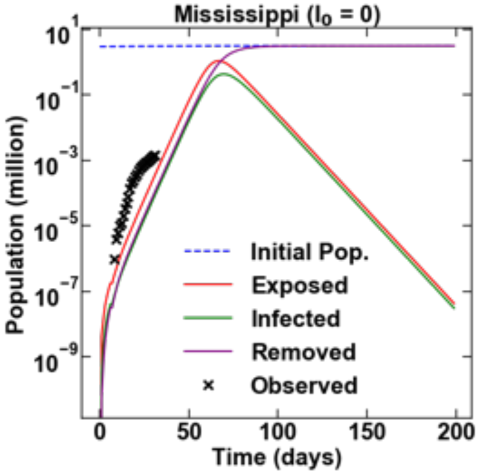

(24)

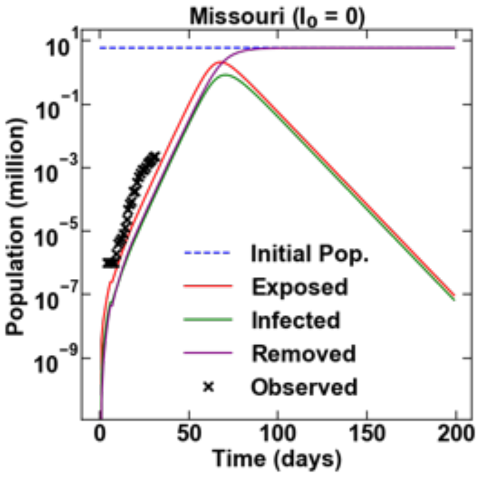

(25)

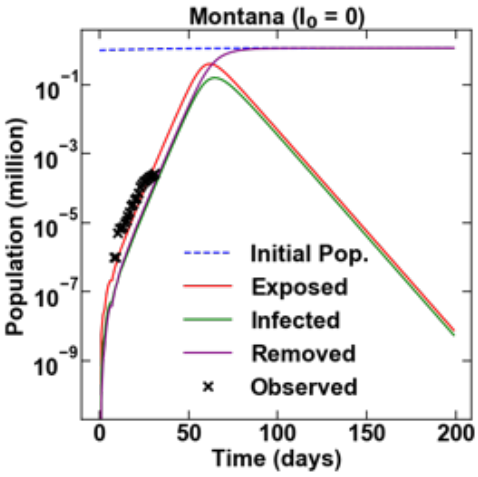

(26)

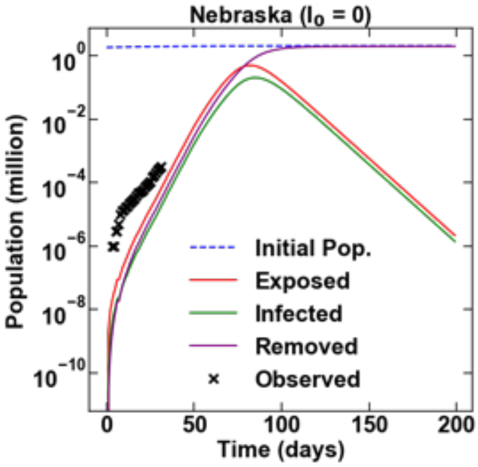

(27)

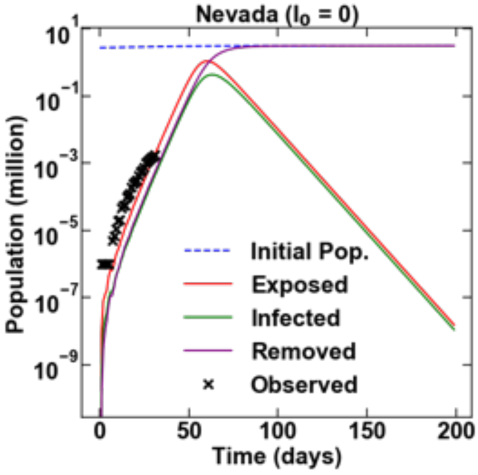

(28)

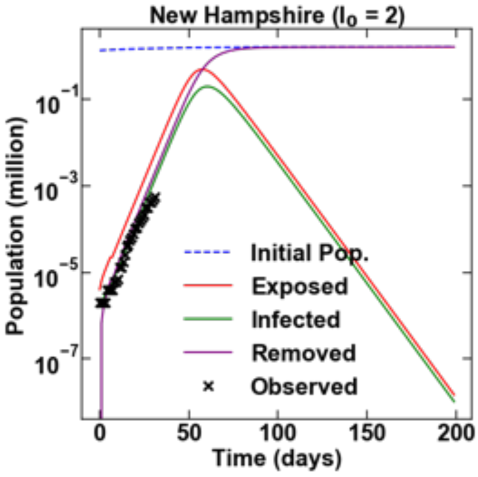

(29)

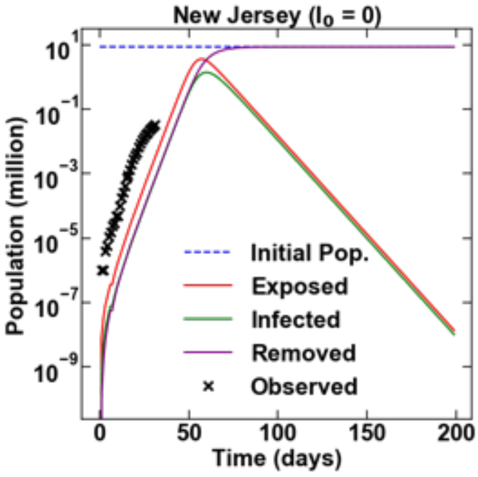

(30)

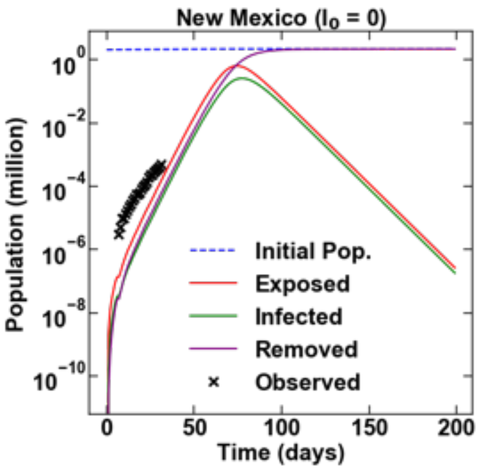

(31)

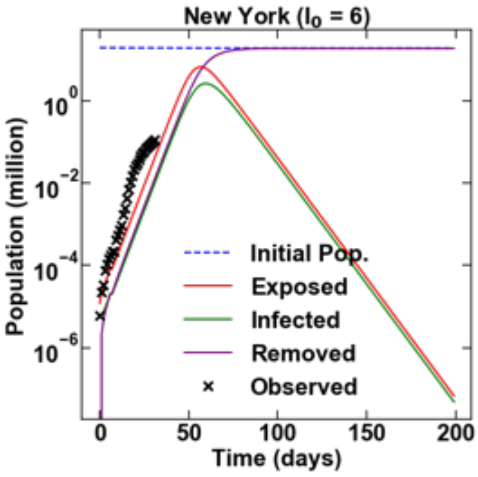

(32)

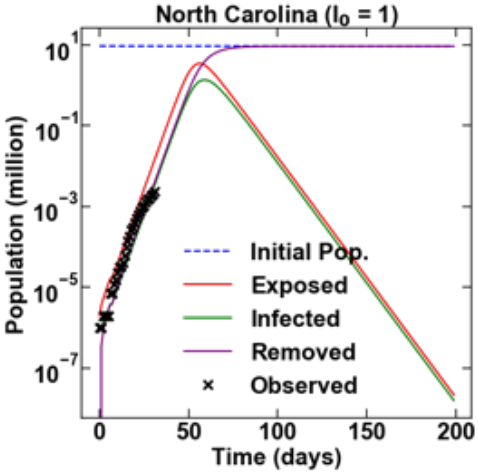

(33)

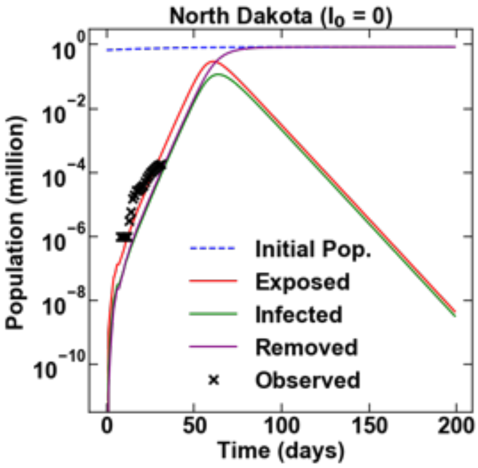

(34)

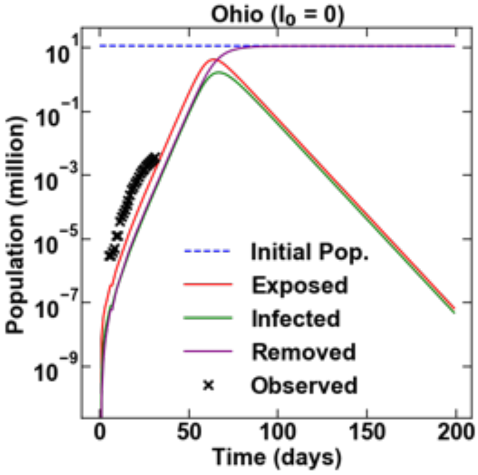

(35)

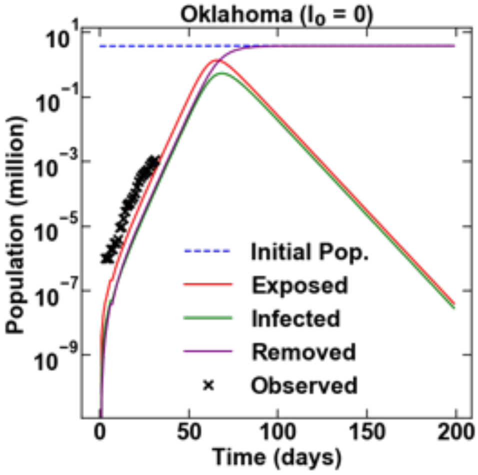

(36)

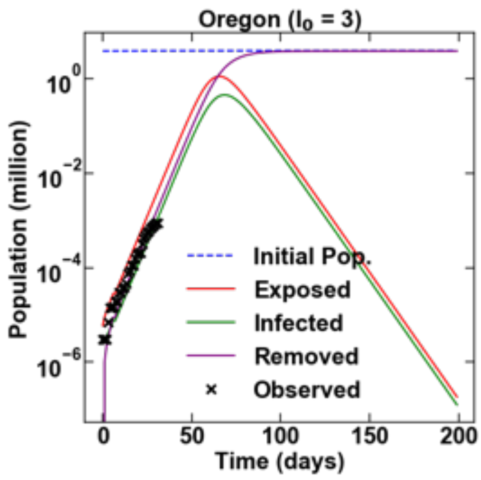

(37)

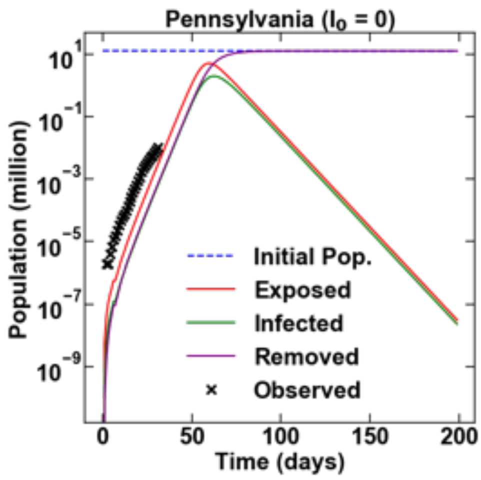

(38)

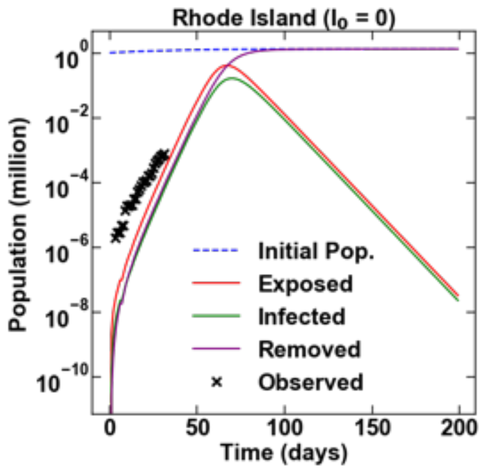

(39)

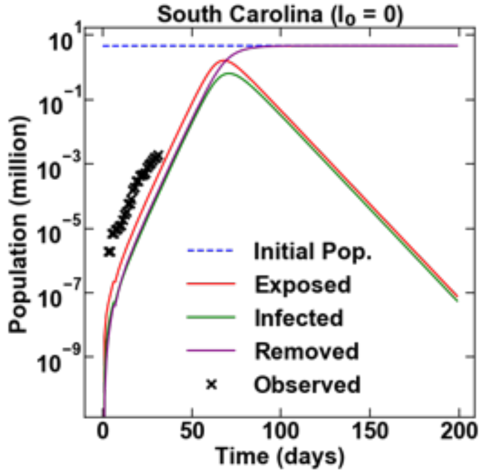

(40)

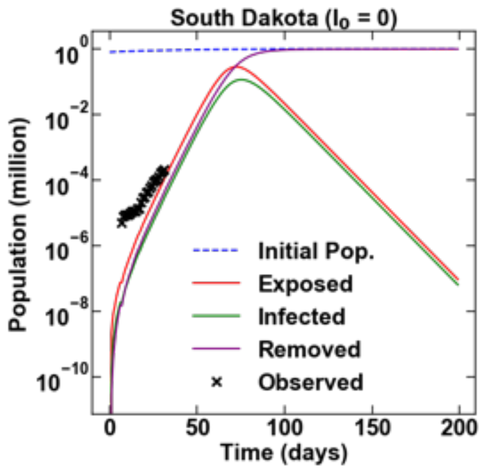

(41)

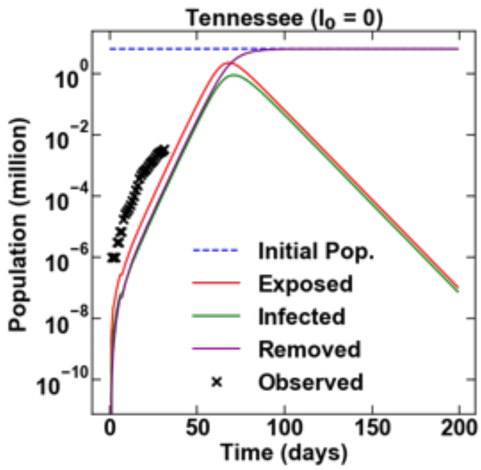

(42)

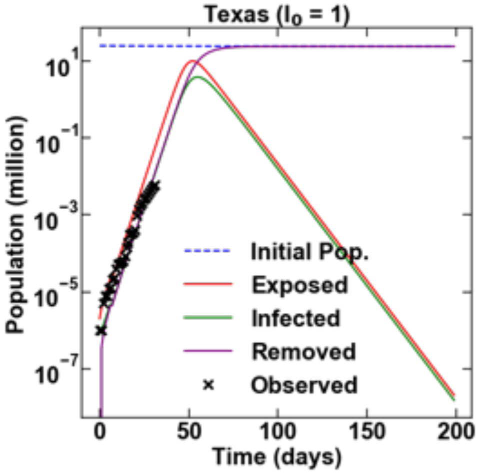

(43)

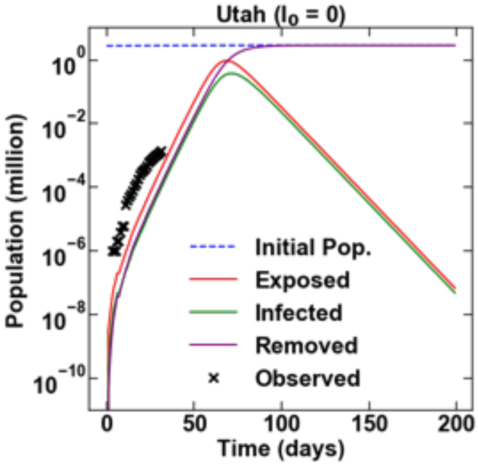

(44)

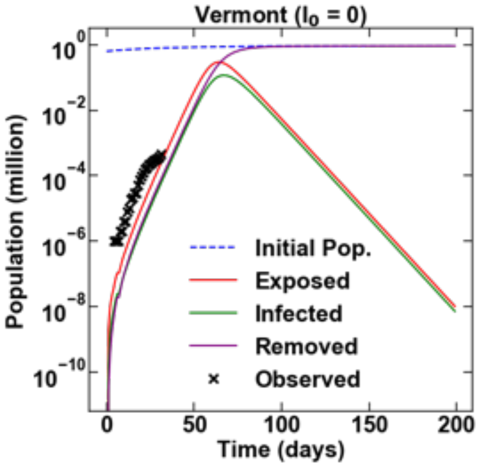

(45)

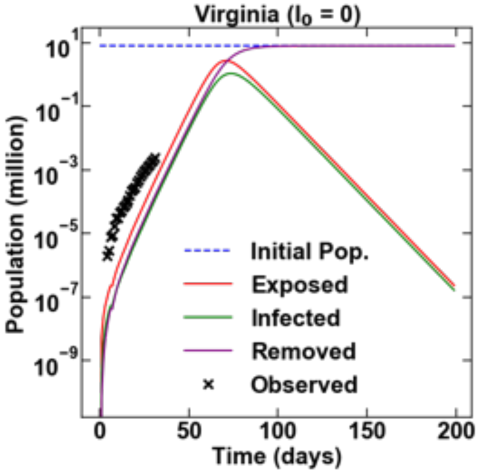

(46)

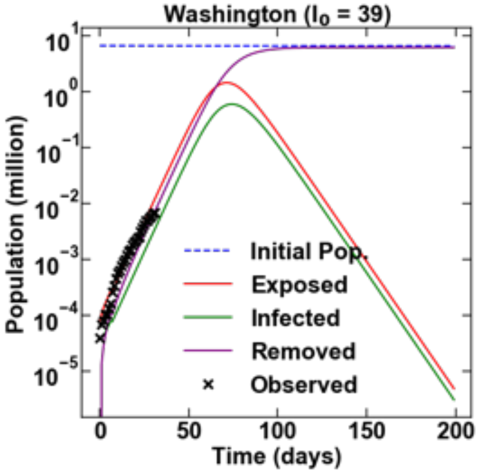

(47)

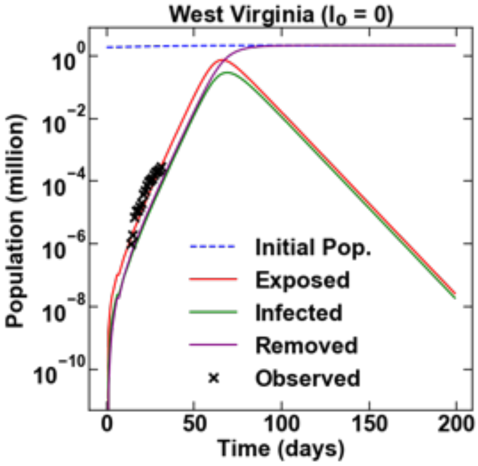

(48)

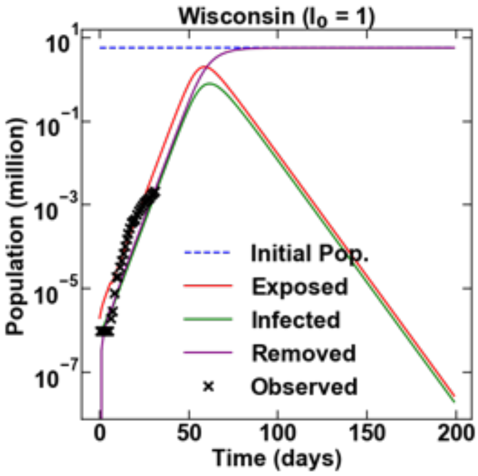

(49)

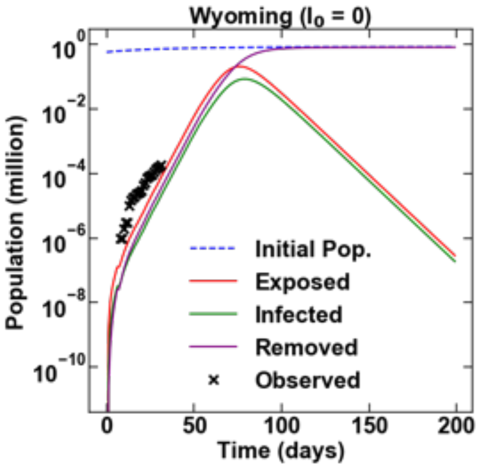

(50)

Fig. S8: Mapping of the pandemic in all the states of USA as predicted by AICSEIR model in comparison to the observed data.

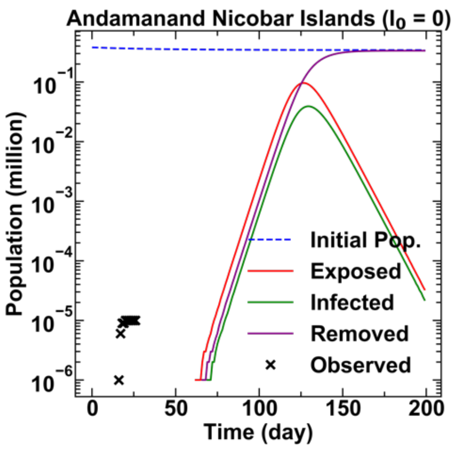
Andamanand Nicobar Islands (I_0 = 0)
Population (million)
Time (day)
Initial Pop.
Exposed
Infected
Removed
Observed

(1)

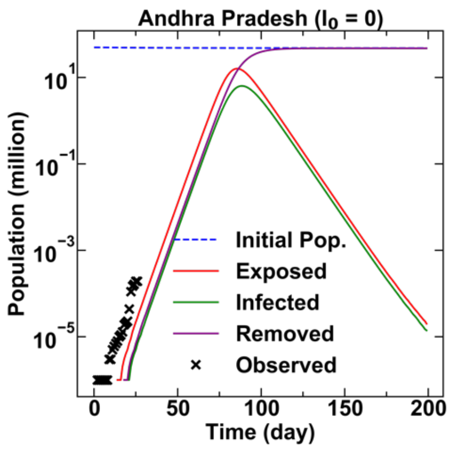
Andhra Pradesh (I_0 = 0)
Population (million)
Time (day)
Initial Pop.
Exposed
Infected
Removed
Observed

(2)

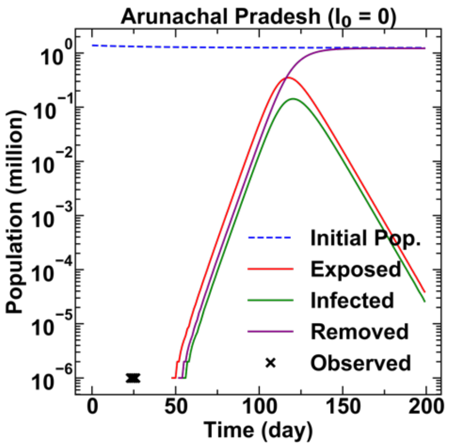
Arunachal Pradesh (I_0 = 0)
Population (million)
Time (day)
Initial Pop.
Exposed
Infected
Removed
Observed

(3)

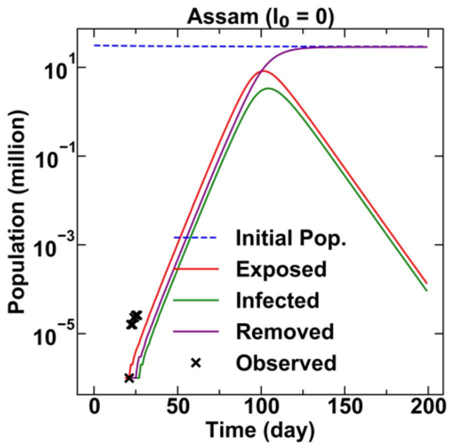
Assam (I_0 = 0)
Population (million)
Time (day)
Initial Pop.
Exposed
Infected
Removed
Observed

(4)

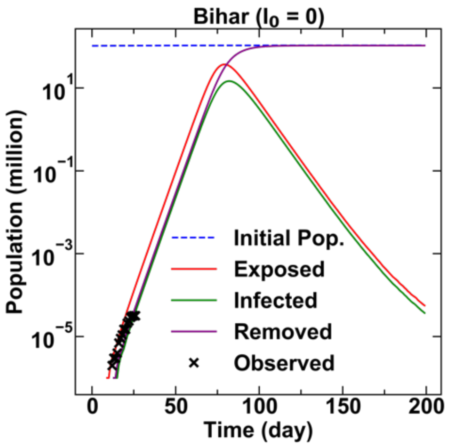
Bihar (I_0 = 0)
Population (million)
Time (day)
Initial Pop.
Exposed
Infected
Removed
Observed

(5)

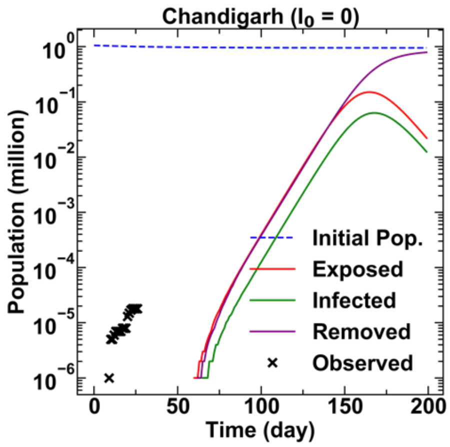
Chandigarh (I_0 = 0)
Population (million)
Time (day)
Initial Pop.
Exposed
Infected
Removed
Observed

(6)

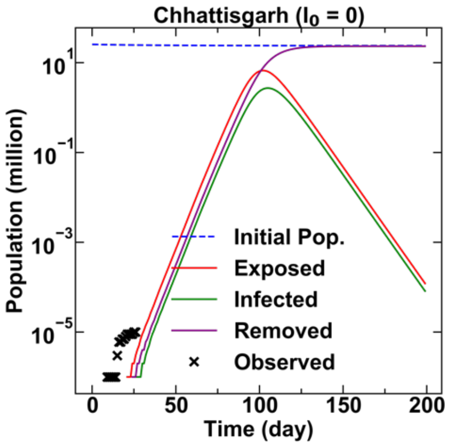
Chhattisgarh (I_0 = 0)
Population (million)
Time (day)
Initial Pop.
Exposed
Infected
Removed
Observed

(7)

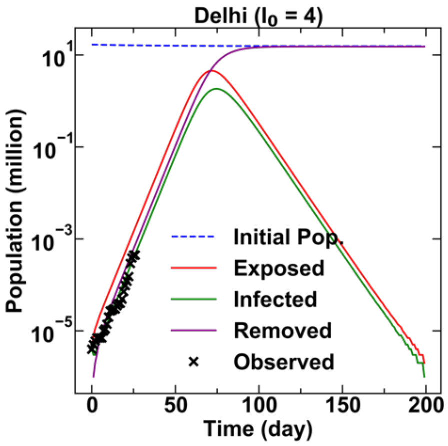
Delhi (I_0 = 4)
Population (million)
Time (day)
Initial Pop.
Exposed
Infected
Removed
Observed

(8)

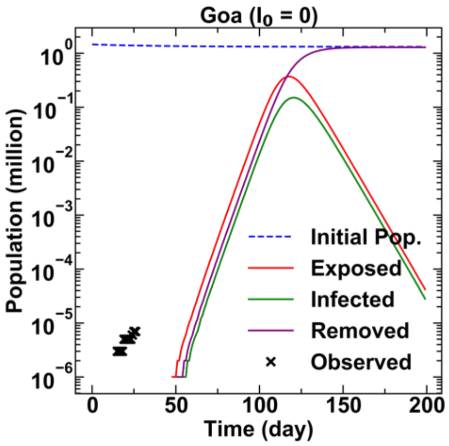
Goa (I_0 = 0)
Population (million)
Time (day)
Initial Pop.
Exposed
Infected
Removed
Observed

(9)

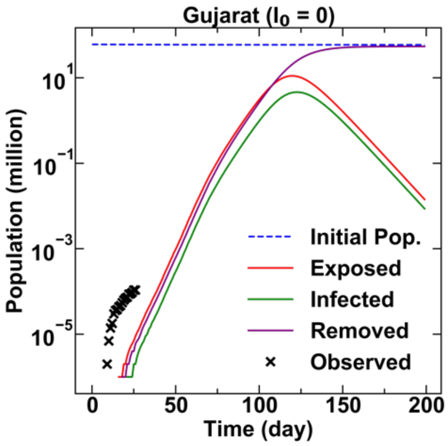
Gujarat (I_0 = 0)
Population (million)
Time (day)
Initial Pop.
Exposed
Infected
Removed
Observed

(10)

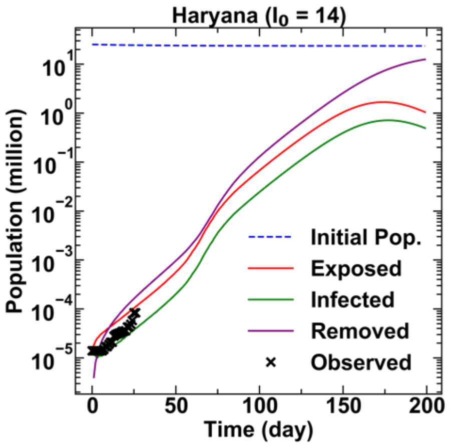
Haryana (I_0 = 14)
Population (million)
Time (day)
Initial Pop.
Exposed
Infected
Removed
Observed

(11)

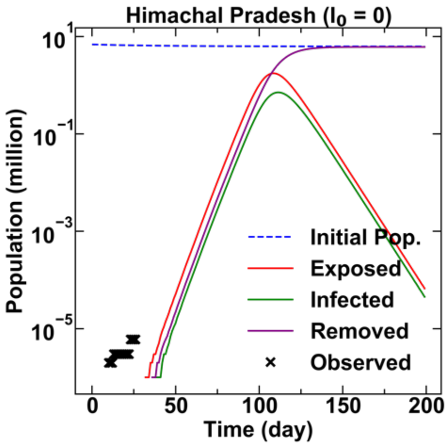
Himachal Pradesh (I_0 = 0)
Population (million)
Time (day)
Initial Pop.
Exposed
Infected
Removed
Observed

(12)

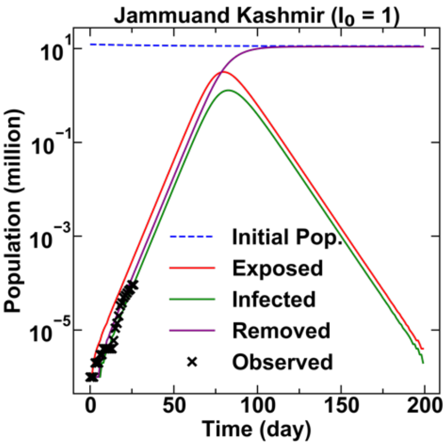

(13)

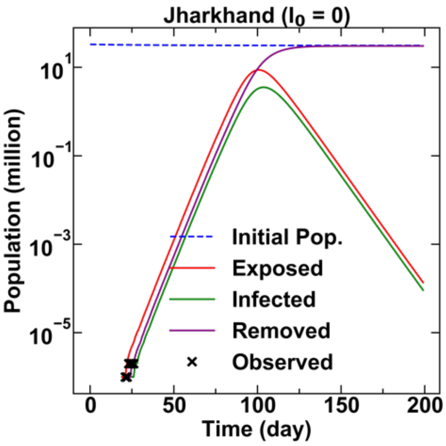

(14)

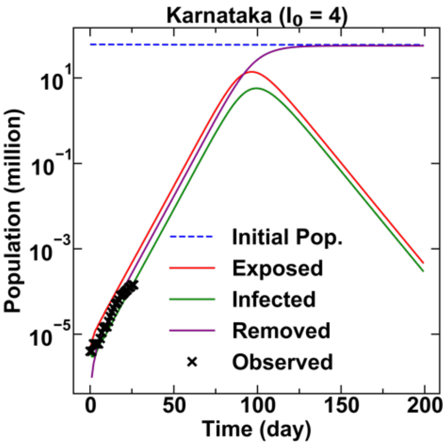

(15)

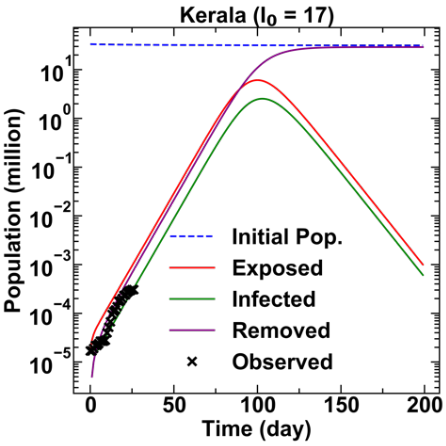

(16)

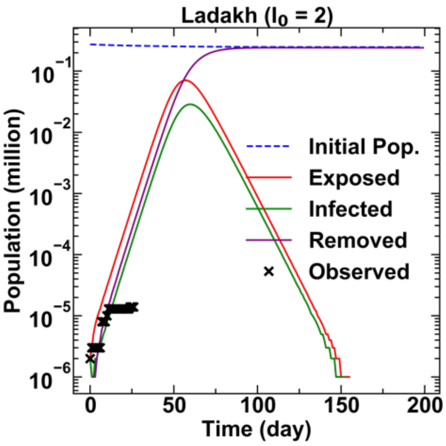

(17)

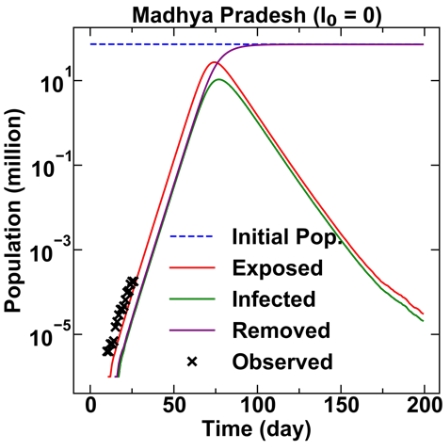

(18)

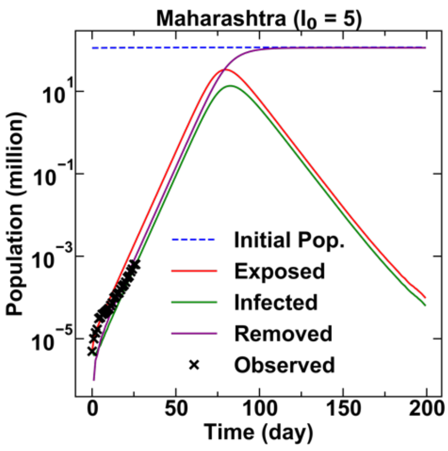

(19)

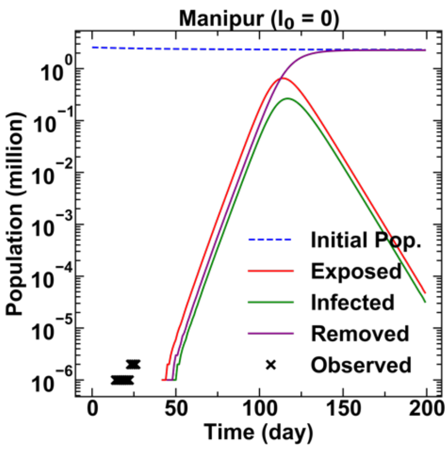

(20)

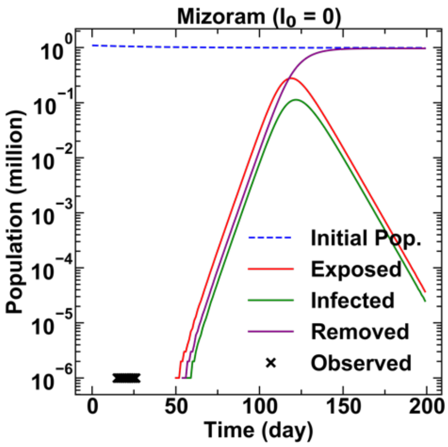

(21)

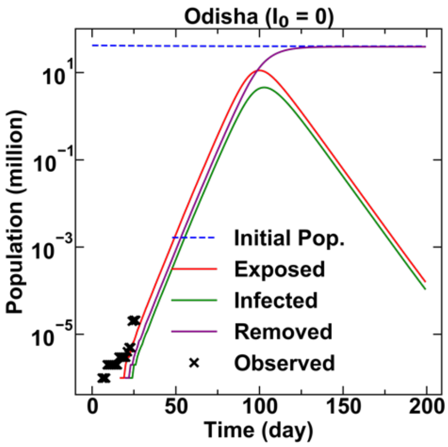

(22)

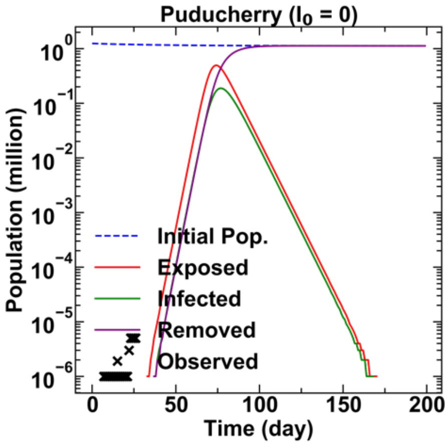

(23)

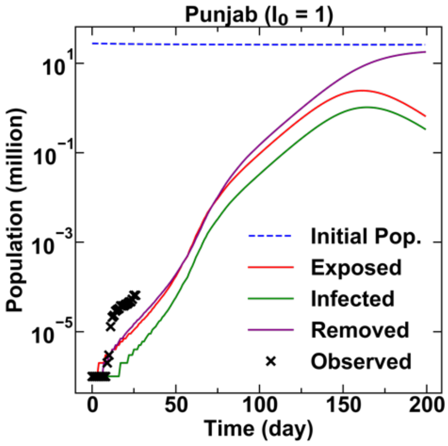

(24)

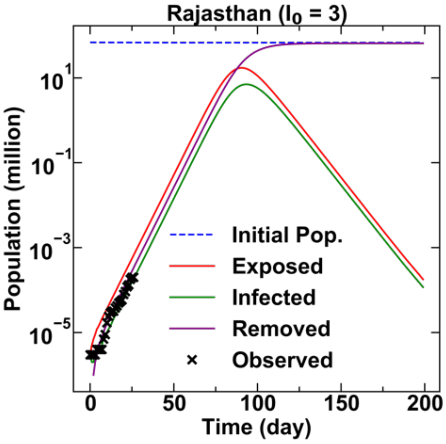

(25)

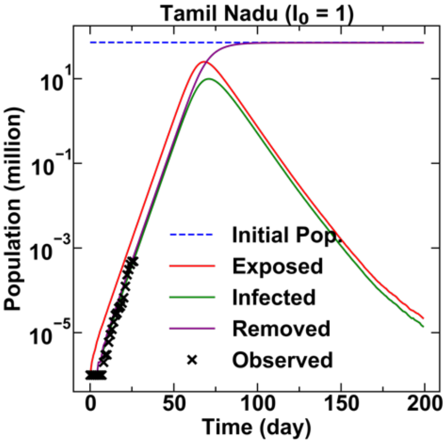

(26)

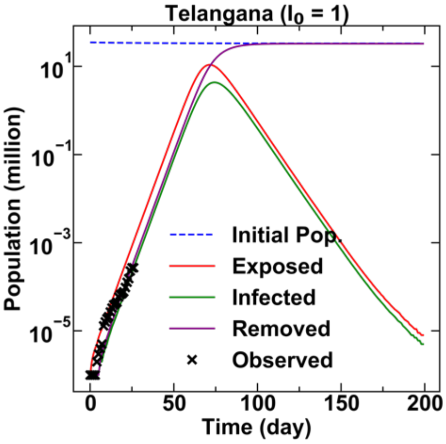

(27)

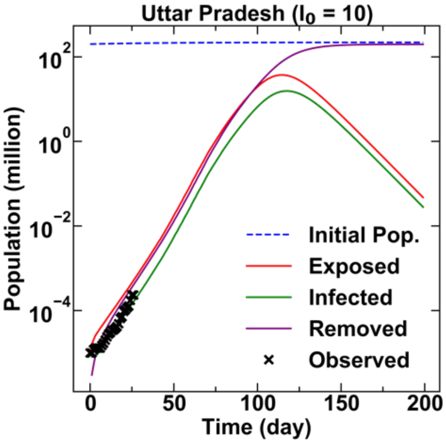

(28)

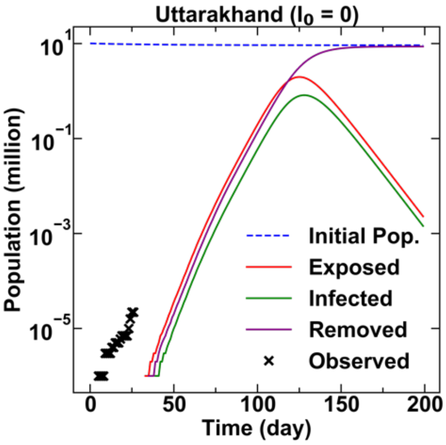

(29)

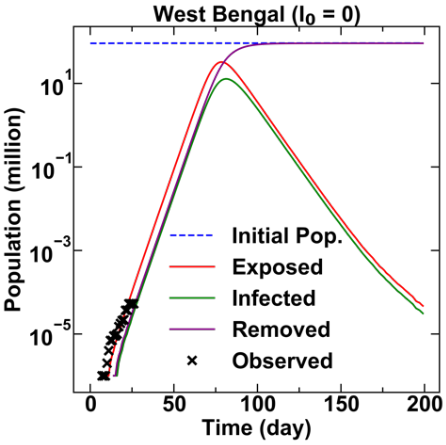

(30)

Fig. S9: Mapping of the pandemic in all the states of India as predicted by AICSEIR model in comparison to the observed data.
